# Visualizing liquid-liquid phase transitions

**DOI:** 10.1101/2023.10.09.561572

**Published:** 2024-10-28

**Authors:** Bikash R. Sahoo, Xiexiong Deng, Ee Lin Wong, Nathan Clark, Harry Yang, Vivekanandan Subramanian, Bryan B. Guzman, Sarah E. Harris, Budheswar Dehury, Emi Miyashita, J. Damon Hoff, Vojč Kocaman, Hirohide Saito, Daniel Dominguez, Janez Plavec, James C.A. Bardwell

**Affiliations:** 1 Howard Hughes Medical Institute; 2 Department of Molecular, Cellular and Developmental Biology, University of Michigan, Ann Arbor, MI-48109, USA; 3 College of Pharmacy, University of Kentucky, Lexington, KY-40508, USA; 4 Department of Pharmacology, University of North Carolina, Chapel Hill, NC-27514, USA; 5 Department of Biochemistry and Biophysics, University of North Carolina, Chapel Hill, NC-27514, USA; 6 Department of Bioinformatics, Manipal School of Life Sciences, Manipal-576104, India; 7 Center for iPS Cell Research and Application, Kyoto University, Kyoto-6068507, Japan; 8 Department of Biophysics, University of Michigan, Ann Arbor, MI-48109, USA; 9 National Institute of Chemistry, Ljubljana, Slovenia

## Abstract

Liquid-liquid phase condensation governs a wide range of protein-protein and protein-RNA interactions in vivo and drives the formation of membrane-less compartments such as the nucleolus and stress granules. We have a broad overview of the importance of multivalency and protein disorder in driving liquid-liquid phase transitions. However, the large and complex nature of key proteins and RNA components involved in forming condensates such as stress granules has inhibited a detailed understanding of how condensates form and the structural interactions that take place within them. In this work, we focused on the small human SERF2 protein. We show here that SERF2 contributes to the formation of stress granules. We also show that SERF2 specifically interacts with non-canonical tetrahelical RNA structures called G-quadruplexes, structures which have previously been linked to stress granule formation. The excellent biophysical amenability of both SERF2 and RNA G4 quadruplexes has allowed us to obtain a high-resolution visualization of the multivalent protein-RNA interactions involved in liquid-liquid phase transitions. Our visualization has enabled us to characterize the role that protein disorder plays in these transitions, identify the specific contacts involved, and describe how these interactions impact the structural dynamics of the components involved in liquid-liquid phase transitions, thus enabling a detailed understanding of the structural transitions involved in early stages of ribonucleoprotein condensate formation.

Although the importance of multivalency and protein disorder in driving the liquid-liquid phase transitions that lead to the formation of membrane-less compartments such as the nucleolus or stress granules is clear,^1^ there is limited knowledge of the structural basis of phase transitions and the conformational changes that occur in macromolecules as they undergo phase transitions.^2^ A better understanding of the molecular mechanisms underlying liquid-liquid phase compartments and their formation should provide valuable insights into the roles of these recently recognized compartments in the cell.^3^

When cells experience stressors that act to dissociate the translation complex such as oxidative or osmotic stress, or viral infection, ribosome run-off occurs, and the resulting naked mRNA recruits proteins to form one liquid-liquid compartment known as stress granules.^4^ Though their exact cellular functions are not yet clearly defined, these particular membrane-less organelles are implicated in sequestering untranslated mRNAs and various RNA-binding proteins, thereby modulating gene expression, and in protein and RNA storage and in protecting cells from stress-induced damage.^5^ Upon stress induction, G3BP1 undergoes a conformational change, enabling it to bind to target mRNAs and recruit other stress granule components, leading to the formation of stress granules.^6^ The G3BP1 protein appears to facilitate stress granule assembly through interactions with other RNA-binding proteins and mRNAs and by promoting the condensation of translationally repressed RNA.^4,7^ The presence of several stress granule proteins such as caprin-1 and TIA1 can influence G3BP1 phase separation,^6^ a process that also likely contributes to stress granule formation. Non-canonical RNA structures known as G- quadruplexes (rG4s) can be recognized and bound by G3BP1,^8^ promoting the recruitment of G4 containing mRNAs to stress granules.^9^ The reversible nature of stress granules ensures that sequestered mRNAs can be promptly released and translated once the stress is alleviated, facilitating the rapid recovery of cellular functions. In summary, the interaction between G3BP1, RNA G4,^8^ and likely other proteins are pivotal for the selective recruitment and sequestration of mRNAs within stress granules.

G4 structures are widely distributed throughout evolution, being particularly common in eukaryotic genomes. These non-canonical nucleic acid secondary structures are four-stranded, and contain stacks of four-guanine planar motifs called G-quartets.^10,11^ G-quartets consist of guanine (G-G) bases that are bonded through Hoogsteen geometry and stabilized by a centrally located monovalent cation, a very different structure than Watson-Crick base paired structures.^12^ Both DNA G4 and RNA rG4 have been detected in vivo.^10,13,14^ rG4 quadruplex-specific antibodies and small-molecule probes have demonstrated the localization of these non-canonical structures in the nucleus, cytoplasm, mitochondria, and the endoplasmic reticulum of living cells.^15–17^ rG4 forming sequences are enriched in untranslated regions of mRNA, ribosomal RNA, pre-mRNA, microRNA, long non-coding RNA, and are present in the non-coding telomeric repeat-containing RNA (TERRA) that is transcribed from telomeric DNA repeats.^18^ Evidence links rG4s to several cellular processes including telomere maintenance, initiation of DNA replication, control of transcription and translation, and genetic and epigenetic instability.^19–23^ Further, expansion of the rG4 forming G4C2 hexanucleotide RNA repeat sequences within the *C9orf72* gene is tightly linked to neurodegenerative diseases that include amyotrophic lateral sclerosis (ALS) and frontotemporal dementia (FTD).^24^ Though rG4s are recently emerging as important players in human transcriptome, they are thought to be only transiently folded.^25^ However, in cells experiencing stress conditions, rG4s are enriched and bind to disordered proteins.^26–29^ Upon binding, they appear to be involved in the formation and organization of stress granules.^24,28,29^

Despite the emerging importance of rG4s, relatively little is known regarding the regulation of their folding and function. Several proteins with disordered domains, including nucleolin,^30^ helicases,^31,32^ FUS,^33^ hnRNPA1,^34^ TDP43,^35^ and TRF2^36^ have been shown to interact with rG4s, regulate G-quadruplex function, and undergo liquid-liquid phase transitions. Helicases, for example, are thought to safeguard against G4-induced genome instability,^37^ while nucleolin acts to enhance the stability of G4 structures.^38^ Determining the mechanism and structural details of protein-rG4 quadruplex interactions will aid in understanding how proteins can regulate the functions of these non-canonical nucleic acid structures. However, our understanding of these interactions is complicated by the often-dynamic nature of the protein and rG4 quadruplex components of these complexes and their tendency to undergo phase transitions.^39^

SERF family proteins were initially identified as in vivo drivers of amyloid formation, a process that has been linked to various age-related diseases.^40–43^ However, their normal physiological functions remain obscure.^43^ SERF related proteins share a conserved, highly charged N-terminal domain and are remarkably small, ~60–80 amino acids in length.^43^ Previously, we showed that the SERF-related human Znf706 protein binds with high affinity to DNA G4 using primarily its conserved N-terminal SERF domain.^44^ Here, we found that the human SERF2 protein specifically binds to rG4s. Through studying these interactions, we are able to visualize the protein-RNA interactions involved in liquid-liquid phase transitions at high resolution.

## RESULTS

### SERF2 is localized to stress granules

**Fig.1:**
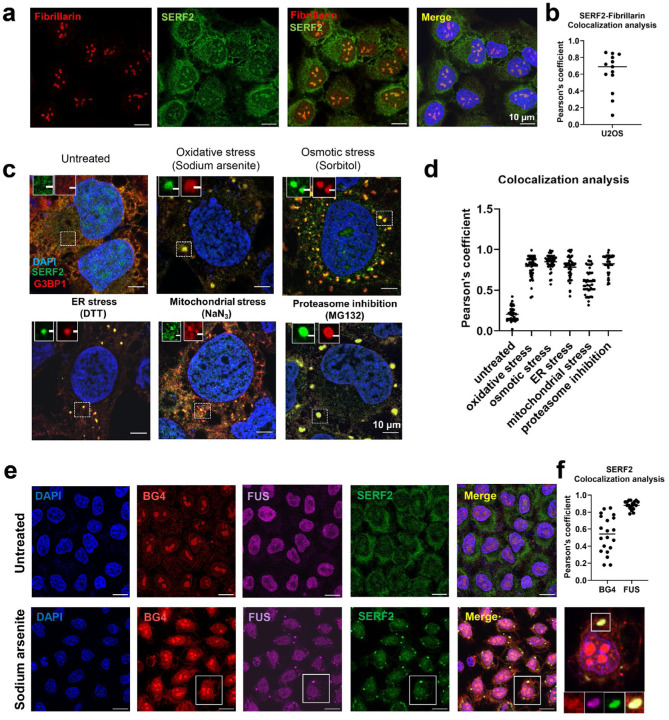
SERF2 colocalizes with stress granules upon various stress conditions. **a,** Immunofluorescence images show that endogenous SERF2 is prevalently distributed in the nucleolus of fixed U2OS cells, as evidenced by fibrillarin staining. **b,** Colocalization analysis plot of fibrillin and SERF2 obtained from **(a)**. **c,** SERF2 forms cytoplasmic foci and co-localizes with the core stress granule marker protein G3BP1 in different stress conditions, suggestive of its involvement in stress granule formation or stability. **d,** The plot shows the quantification of stress granules retrieved from **(c)** under various stress conditions containing both SERF2 and G3BP1. **e,** Fixed U2OS immunofluorescence cell images showing oxidative stress-induced granules containing SERF2, FUS, and rG4 quadruplexes detected by specific antibodies indicated in green, purple, and red, respectively. **f,** Plot showing SERF2 colocalization with FUS and rG4 quadruplexes retrieved from **(e)** as a measure of Pearson’s coefficient. The scale bars in **(a, c, and e)** are 10 μm.

SERF family of proteins are rich in the amino acids E, D, R, and K, from whence comes their name, Small EDRK-Rich Factor.^43^ SERF proteins were initially isolated for their ability to accelerate amyloid formation in a *C. elegans* model of Huntington’s disease.^40^ Under normal growth conditions, we find that the human SERF2 protein is partially localized to the nucleus, diffusely staining in the cytoplasm, but is most enriched in the nucleolus, a membrane-less liquid-liquid phase separated compartment (Fig. 1a, b). This localization is reasonably consistent with a previous study that found the homologous protein SERF1a normally localizes to the nucleus but is exported into the cytosol upon nucleolar stress.^45^ Following up on that latter observation, we found that upon treatment with the oxidative, osmotic, endoplasmic reticulum, mitochondrial, and proteasome stressors (Fig. 1c), but not heat stress (Supplementary Data Fig. 1a), prominent SERF2 foci were observed in the cytoplasm (Fig. 1d). We determined that SERF2 colocalizes with five different stress granule marker proteins (Fig. 1e and Supplementary Data Fig. 1b) making SERF2 a component of the stress granule, which like the nucleolus, is also a liquid-liquid phase compartment.^46^

### SERF2 is involved in stress granule formation

**Fig. 2|:**
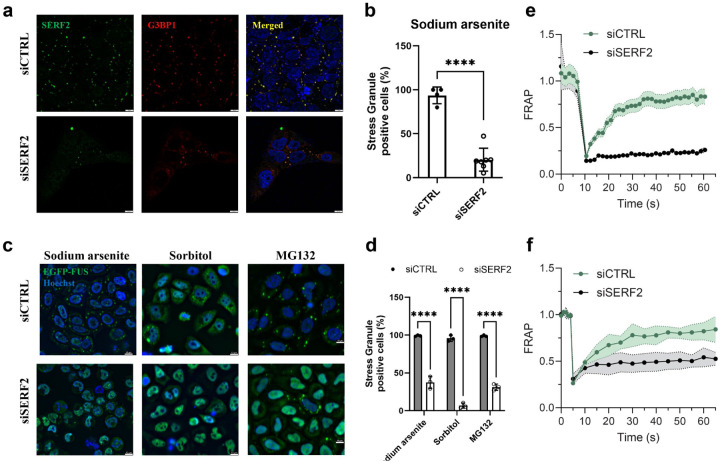
SERF2 regulates stress granule formation and dynamics. **a,** Immunofluorescence of SERF2 and G3BP1 in fixed U2OS cells treated with 0.5 mM sodium arsenite for 1 hour. **b,** Plot shows percentage of stress granule positive cells under sodium arsenite treatment calculated from images shown in (**a**). Error bars were calculated from four independent experiments. **c,** Live-cell imaging of EGFP-FUS HeLa Kyoto cells with siCTRL or siSERF2 conditions, treated with different stressors (Sodium arsenite, 0.5 mM; Sorbitol, 0.4 M; MG132, 10 μM) for 1 hour. Scale bars in (**a** and **c**) are 10 μm. **d,** Plot shows percentage of stress granule positive cells under different stress treatments calculated from images shown in (**c**), **** shown in (**b** and **d**) indicates P < 0.0001. **e,f,** The graphs show FRAP recovery curves in EGFP-FUS HeLa Kyoto cells with siCTRL or siSERF2 conditions treated with 0.4 M sorbitol and (**e**) 0.5 mM sodium arsenite (**f**).

We next investigated if SERF2 is involved in nucleolar or stress granule occurrence. To test this, we knocked down SERF2 using RNAi in oxidative stressed U2OS and BJ fibroblast cells. We monitored nucleoli using DAPI staining of the nuclei, where nucleoli appear as distinct dark patches and stress granules using an antibody to G3BP1, a core stress granule marker protein that is thought to be key in stress granule formation.^6,47^ Nucleolar size and shape appeared to be normal in SERF2 knockdown cells as observed by antibody staining with the nucleolar marker, Nopp140 (Supplementary Data Fig. 1c). Sodium arsenite triggered the formation of stress granules as expected.^48^ 94% of control siRNA injected cells were stress granule positive, however, in cells that had been depleted of SERF2, much more diffuse G3BP1 staining was observed, and quantification showed that only 20% of these SERF2 knockdown cells were stress-granule positive (Fig. 2a, 2b, and Supplementary Data Fig. 2a). A CRISPR-Cas9 knockout of SERF2 in HEK293T cells also had a much smaller percentage of stress granule positive cells than control wild-type cells (Supplementary Data Fig. 2b). We also observed a decrease in the number of stress granules when SERF2 was knocked down in live HeLa Kyoto cells bearing the stress granule marker EGFP-FUS, upon sodium arsenite, sorbitol, or MG132 treatments, all of which are known to induce stress granule formation (Fig. 2c, 2d). A SERF2 knockdown resulted in a similar reduction of EGFP-FUS stress granules that were formed in response to arsenite stress as compared to a double knockdown of G3BP1 and G3BP2 (Supplementary Data Fig. 2c). Individual knockdowns of G3BP1 or G3BP2 failed to result in a significant reduction in EGFP-FUS puncta. These results indicate that SERF2 may play as important a role in stress granule formation or stabilization as the combination of the G3BP1 and G3BP2 proteins do.^6^ Stress granules have been shown to possess dynamic, reversible, and fluid-like properties.^49^ Therefore, we next assessed the stress granule properties in cells depleted with SERF2 and compared them to cells treated with a control siRNA by fluorescence recovery after photobleaching (FRAP) measurements. The stress granules formed by sorbitol treatment in control siRNA treated U2OS cells, recover the majority of their original fluorescence after fluorescence photobleaching within 60 seconds (Fig. 2e), the fewer, and smaller granules produced in SERF2-depleted U2OS cells show almost no recovery within 60 seconds. A similar though less dramatic result is seen for stress granules formed by sodium arsenite treatment (Fig 2f). These results indicate that SERF2 is not only impacting the size and distribution of stress granules but also altering their ability to recover after photobleaching. Our data suggests that SERF2 promotes stress granule formation and may enhance their fluidity.

### SERF2 binds RNA G-quadruplexes

**Fig. 3|:**
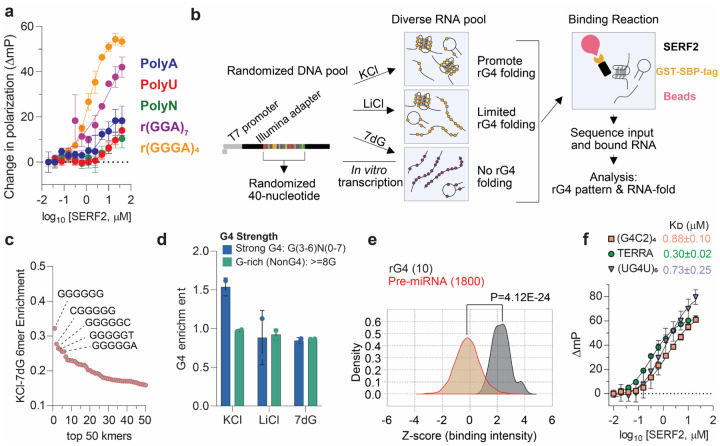
High-throughput screening of SERF2 binding substrates. **a,** Fluorescence polarization assay measuring the binding affinity of SERF2 with 6-FAM labeled random ribo-polynucleotides and rG4 quadruplex forming sequences as indicated. **b,** A schematic representation of RNA bind-n-seq experiments with RNA pools. A randomized DNA oligo was transcribed to RNA and folded in buffer containing KCl or LiCl. An additional RNA pool was made by replacing guanines (G) with 7-deaza (7dG) to limit rG4 quadruplex folding. These pools were mixed with GST-SBP-SERF2, and bound RNA was isolated and sequenced to ~10–20+e6 reads. **c,** RNA bind-n-seq analysis of the 6-mers enrichment in KCl versus RNA made with 7dG in a sample mixture containing 50 nM SERF2. The guanine-rich 6mers in the top 5 kmers are labeled. **d,** Enrichment of rG4 quadruplex patterns in different conditions and varied G4 quadruplex strengths containing 50 nM SERF2. Sequences containing 3 or more guanines in the G-tetrad are referred to as strong G4 quadruplexes, while sequences with ≥8 guanines and lacking a defined G4 forming motif are referred to as non-G4 quadruplexes. **e,** FOREST analysis average binding intensities of SERF2 containing 1800 folded human pre-miRNA and 10 rG4 quadruplex structures. The p-value was determined by the two-tailed Brunner-Munzel test. **f,** Fluorescence polarization plot shows the binding affinity between SERF2 and three different 6-FAM labeled rG4 quadruplexes measured at room temperature. The binding assays in **a** and **f** were done with varied protein concentrations mixed with 20 nM rG4 quadruplex or polynucleotides at 20 nM in 20 mM NaPi (pH 7.4) and 100 mM KCl.The standard deviations are calculated from three independent replicates.

Stress granules are enriched with both RNA and ribonucleoproteins, and recent evidence shows the enrichment of rG4s, non-canonical tetrahelical structures within these compartments.^27,29,50^ We recently showed that the SERF2 homologous protein, human Znf706, binds specifically to G-quadruplexes primarily through a conserved N-terminal disordered SERF domain.^44^ Previously, the human SERF1a protein was shown to bind to an unstructured 21 nucleotide RNA.^45^ Though this reported interaction was very weak, with a binding affinity (K_D_) of ~5 μM in unphysiologically low salt conditions (20 mM NaCl), we wondered if the related protein human SERF2 can also interact, at least weakly, with RNA. Using fluorescence polarization binding assays conducted under the more physiological salt conditions of 20 mM sodium phosphate and 100 mM KCl, SERF2 failed to bind with polyA, polyC, and polyU homo-polyribonucleotide sequences, and single or double-stranded RNA sequences containing hairpin structures (Fig. 3a, Supplementary Data Figs. 3a, 3b). However, we observed that polyG, (GGGA)_4_, (GGA)_7_, (G4C2)_4_ and (AGG)_5_ repeat RNA sequences which are known to form G-quadruplex structures,^9,51^ do bind to SERF2 with sub-micromolar binding affinities (Figs. 3a, and Supplementary Data Fig. 3). To get a preliminary impression as to whether SERF2 specifically binds these non-canonical structures, we tested its interaction with two RNAs with similar sequences: (AGG)_5_, which is known to fold into an rG4,^9^ binds SERF2 with a K_D_ of 1.8 ± 0.5 μM, and (ACG)_5_, that does not form G4 quadruplexes shows no measurable binding (Supplementary Data Fig. 3c) suggestive of SERF2 having a binding preference for rG4 structures.

We found SERF2 to be predominantly localized in the nucleolus, an RNA-rich liquid-liquid phase compartment, and we also found it to be present in the nucleus, a compartment in which both RNA and DNA G-quadruplexes exist. The SERF homolog Znf706 has been identified to bind DNA G-quadruplexes.^44^ We decided to test SERF2’s relative affinity to RNA and DNA G-quadruplexes. A key difference between RNA and DNA G-quadruplexes is that rG4s are largely confined to those with a parallel topology, while DNA G-quadruplexes can adopt parallel, antiparallel, and hybrid topologies.^52^ We used two pairs of G-quadruplexes formed from pairs of identical DNA and RNA sequences and the telomeric repeat DNA and RNA sequence. A similar binding affinity was observed for interactions between SERF2 and a model DNA 4-repeat GGGA sequence as compared to its counterpart GGGA repeat RNA sequence (Supplementary Data Fig. 4a). Both DNA and RNA 4-repeat GGGA species form very similar G-quadruplex structures of parallel topology (Supplementary Data Fig. 4b). On the other hand, fluorescent polarization binding assays demonstrated >10-fold and >20-fold stronger binding of SERF2 to the 4-repeat hexanucleotide G4C2 RNA expansion repeats, and telomeric repeat RNA TERRA rG4 quadruplexes as compared to their DNA G-quadruplex counterparts (Supplementary Data Fig. 4c, d). As is commonly observed, these RNA sequences fold into G-quadruplexes of parallel topology, whereas their DNA counterparts fold into a mixed topology.^53,54^

### RNA binding sequence specificity of SERF2

Since it appears that SERF2 tends to interact more strongly with rG4s than their DNA counterparts, we decided to explore SERF’s RNA binding specificity in more detail. We utilized two independent high-throughput screening methods, RNA bind-n-seq^55^ and FOREST^56^ (Folded RNA Element profiling with STructure). Both methods have previously been validated using structured and unstructured RNA molecules, including those that form rG4s, and known RNA binding proteins including those that specifically bind to rG-quadruplexes.^55,56^ RNA bind-n-seq, as illustrated in Fig. 3b, uses a diverse RNA pool generated by transcribing a randomized DNA library. The RNA species bound to SERF2 were isolated using RNA pools folded in three different buffer conditions, one containing KCl, known to promote G-quadruplex folding, a second containing LiCl, which disfavors G-quadruplex formation,^57^ and the third synthesized using 7-deaza guanine in place of guanine,^58^ which eliminates G-quadruplex formation (Fig. 3b). Features of RNAs that are associated with SERF2 binding were identified by determining k-mer (k=6) enrichments.^55^ We assessed k-mers preferentially enriched in KCl conditions versus k-mers enriched from the 7-deaza RNA pool. The top five 6-mers specifically enriched in KCl are all very rich in guanines and were predicted to form stable G-quadruplexes by the quadruplex forming G-rich sequences (QGRS) mapper tool (Fig. 3c).^59^ Due to the massive RNA complexity of the RNA bind-n-seq pools, we were also able to search for sequences predicted to form stable rG4 structures and compare their binding to G-rich sequences that are not predicted to form G-quadruplex structures. Sequences predicted to fold into rG4 structures that contained the canonical sequence motif G_(3–6)_N_(0–7)_ were identified, and non-G4 forming G-rich sequences were defined as those containing ≥ 8 guanines, but not representing a sequence motif G_(3–6)_N_(0–7)_. We noted that sequences predicted to fold into rG4 structures were ~1.5-fold enriched for SERF2 binding in KCl, on the contrary, non-G4 forming G-rich sequences were not enriched in the binding assay (Fig. 3d, Supplementary Data Fig. 4e). Furthermore, no G-quadruplex enrichment was observed in the other binding reactions containing LiCl or RNA pools made with 7-deaza, both known to disrupt rG4 structures (Fig. 3d). These data support a model wherein SERF2 has a binding preference for RNA sequences predicted to fold into stable G-quadruplex structures.

To further probe if SERF2 has a binding preference for rG4s, SERF2 was used to screen an RNA structure library using the FOREST protocol.^56^ This approach was chosen because the ~1800 human-derived sequences in the FOREST library^56^ provide a diverse variety of folded RNA structures to screen for SERF2 binding. The FOREST analysis identified a UGGGGU (UG4U)_6_ repeat rG4 forming sequence^60^ as the top binding partner for SERF2 in this library. This sequence along with 7 other sequences in the top 10 binding hits were predicted to be G-quadruplex forming sequences as tested using the QGRS mapper tool.^59^ Overall, the binding scores of SERF2 for the sub-library experimentally known to form G-quadruplex were statistically significantly higher (4.12 × 10^−24^) than for the other 1800 sequences in the library (Fig. 3e). The significance of the binding differences of SERF2 for the rG4s, as compared to the rest of the library, correlates to that observed previously with a binding score of 8.8 × 10^−41^ and 1.4 × 10^−5^ for the rG4 BG4 antibody and cold-induced binding protein, respectively.^56^

We next measured the in vitro binding affinity of SERF2 to the FOREST hit 6-repeat UG4U sequence, and four additional rG4 quadruplex forming sequences. These were the TERRA repeat-containing RNA (UAGGGUUAGGGUUAGGGUUAGGG),^61^ found in human telomeres, and the four-repeat (GGGGCC)_4_ sequence (referred to as G4C2 hereafter), a hexanucleotide repeat found within the *C9orf72* gene, whose expansion is associated with Amyotrophic Lateral Sclerosis (ALS),^62^ and to the BG4 antibody-bound mRNA G-quadruplexes containing genes *Mark2* (5′-GAAGGGGAGGGGGCUGGGGGGGGGCAGGG-3′) and *Stxbp5* (5’-GGGAAGGGAAGGGGAGUGGG-3’). These latter four quadruplex sequences were picked because they are all known components of stress granules.^24,29,63^ Binding assays showed SERF2 binds to all of these sequences with low micromolar affinity, with K_D_ values for G4C2: 0.88±0.1 μM, TERRA: 0.30±0.02 μM, UG4U: 0.80±0.3 μM, and *Mark2* rG4 4.5±0.5 μM (Fig. 3f and Supplementary Data Figs. 4f,g). Combined, these results indicate that SERF2 interacts strongly with rG4 forming sequences, including some that are present in stress granules.

rG4s are enriched under stress conditions within stress granules.^27,28^ This observation inspired us to test if SERF2 and rG4 are co-localized in cells under stress conditions. We confirmed that rG4s are present in stress granules and we in addition found that under stress conditions, SERF2 co-localizes significantly with the G4 antibody BG4 (Fig. 1c). It is important to point out that G4s are not just present within stress granules but in multiple other cellular compartments as well. This likely explains the lower Pearson’s coefficient of SERF2 localization with BG4 antibody in comparison to the FUS protein which is largely localized to stress granules following stress treatments (Fig. 1f).^64^ Our demonstration that SERF2 is located in stress granules, colocalizes with rG4 (Fig. 1c), and interacts specifically with rG4 in vitro, let us postulate that SERF2 and rG4s may interact in vivo. However, understanding the interaction of SERF2 and rG4s within stress granules is complicated by the presence of hundreds of other proteins and RNA species within these cellular bodies.

### SERF2 forms liquid-liquid phase droplets with RNA G-quadruplexes

**Fig. 4|:**
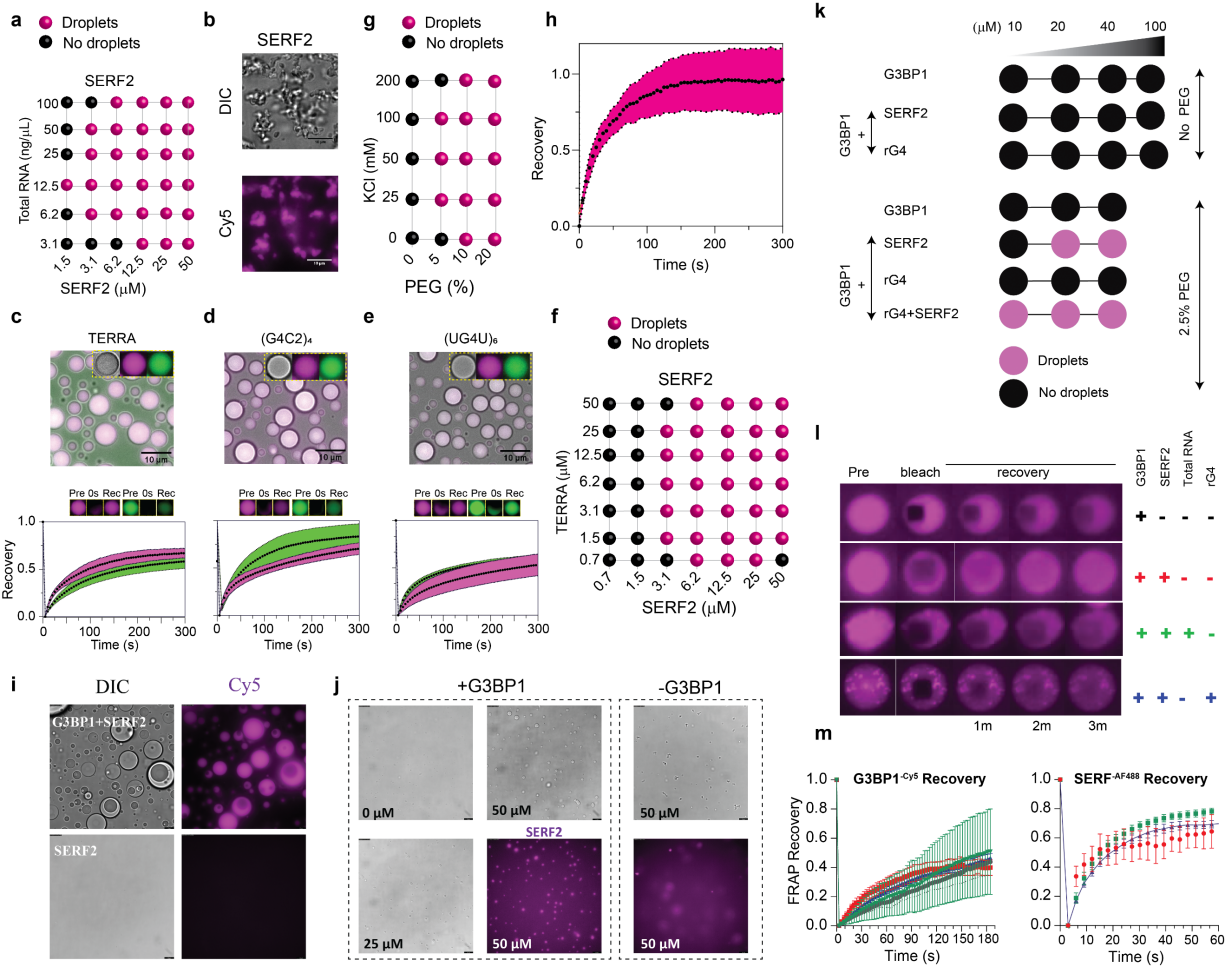
RNA interaction drives liquid-liquid phase transition in SERF2. **a,** Phase regimes illustrating phase transition in SERF2 as a function of total RNA concentration extracted from HeLa cells. **b,** Fluorescence imaging shows gel-like structures in 50 μM SERF2 mixed with 200 ng/μL of total RNA containing 10% (w/v) PEG8000, incubated for 30 minutes at room temperature. **c-e,** 50 μM SERF2, dissolved in 20 mM NaPi (pH 7.4) and 100 mM KCl, readily undergoes a phase transition (**c-e**, top) when mixed with equimolar concentrations of different rG4 quadruplexes, that include TERRA, (G4C2)_4_, and (UG4U)_6_. The sample mixture contains 1/200^th^ Cy-5 labeled SERF2 (purple signals) and 6-FAM rG4 quadruplex (green signals), as indicated in the figure inset. Two-component FRAP analysis (**c-e**, bottom) was done to measure the recovery rates of SERF2 and rG4 quadruplex in the SERF2-rG4s droplets. On the top of each FRAP plot, the pre-bleached, after-bleached (0 s), and recovered droplets (300 s) are shown. The FRAP data were fitted in GraphPad Prism, using a non-linear regression, one-phase association model, to obtain the recovery halftime (t1/2). **f,g,** Schematics showing SERF2 and TERRA rG4 sample mixtures phase separation, in 10% PEG8000, at varied protein to RNA concentrations **(f),** and salt to PEG8000 concentrations **(g)**. **h,** Dynamics and recovery of the Cy5-labeled proteins, in SERF2-total RNA droplets, obtained by FRAP analysis suggesting that the gel-like structures are dynamic and reversible. Standard errors were calculated by analyzing 8 isolated droplets subjected to FRAP. **i,** DIC and fluorescence images showing co-phase separation of SERF2 (purple) and G3BP1 with 12.5 ng/μL HeLa total RNA. **j,** DIC and fluorescence images show SERF2 facilitates G3BP1-RNA condensation in samples containing variable SERF2 and G3BP1 concentrations as indicated and 12.5 ng/μL HeLa total RNA. **k**, Phase diagram showing G3BP1 phase transition in non-crowding and crowding conditions containing PEG8000 in 20 mM NaPi, pH 7.4, 100 mM KCl buffer. G3BP1 phase transition was measured in the absence or presence of SERF2 or a 36-nucletide long UG4U6 rG4 quadruplex or mixture of SERF2 and rG4s as indicated. **l,** Time-series images of 50 μM G3BP1 condensates after photobleaching in the absence or presence of SERF2, total RNA, and TERRA rG4 quadruplexes prepared in 20 mM NaPi, 100 mM KCl (pH 7.4) containing 5% PEG8000. **m,** FRAP recovery plots of G3BP1-Cy5 (left) and SERF2-AF488 (right) obtained from (**l**), and the graph colors correspond to the sample mixture shown in (**l**).

Disordered regions in proteins are often associated with the protein-RNA interactions that drive phase transition in vitro and membrane-less cellular compartmentalization in vivo.^65–67^ In addition to protein disorder, the RNA secondary structure is important in regulating phase transitions.^68^ It has recently been shown that rG4s can mediate liquid-liquid phase transitions.^33,69^ Therefore, we decided to investigate if SERF2 binding to rG4s can also mediate phase transitions. In isolation, SERF2 remains soluble at high concentrations (even at 1 mM) in various salt concentrations (0–200 mM KCl) even under crowding conditions (20 % PEG8000) (Supplementary Data Fig. 5a, 5b). Under non-crowding conditions, fluorescence microscopy also showed no phase separation for SERF2 when mixed with total HeLa cell RNA or rG4s (Fig. 4a, Supplementary Data Fig. 5c, 5d). However, in the presence of small amounts of crowding agent PEG8000, SERF2, even when present in concentrations as low as ~1.5 to 3 μM, phase separates when mixed with total HeLa cell RNA or each of three different rG4 forming sequences TERRA, (G4C2)4, and (UG4U)6 (Fig. 4a, b, Supplementary Data Fig. 5c). This concentration of SERF2 is below the concentration present in different human cells such as 48 ppm (~ 7 μM) in U2OS cells and 163 ppm (~ 23 μM) in MCF7 breast cancer cells in the crowded in vivo environment.^70^ All rG4 forming sequences do not form droplets (Supplementary Data Fig. 5e) on their own in crowding conditions. Our data thus suggests that in vitro SERF2 co-phase separates with rG4 in a sequence-independent manner (Fig. 4c–e).

We next decided to focus on studying the principles of liquid-liquid phase transitions from a structural standpoint using just two macromolecular components, the TERRA rG4 and SERF2. TERRA rG4 was picked because it has been previously structurally well characterized,^71,72^ and because of the evidence for its presence in stress granules, and its potential involvement in their formation and organization.^63^ SERF2 was picked because of its involvement in stress granule formation, and because of its small size and biophysical amiability. We found that SERF2-TERRA droplets were formed at a variety of concentrations of salt, PEG8000, protein, and RNA (Figs. 4f, g, Supplementary Data Fig. 5a). To examine if similarly sized RNA oligomers that fail to form rG4 could result in phase transitions, we tested 10- and 20-nucleotide polyA sequences that do not form rG4, and failed to phase separate in the presence of SERF2 (Supplementary Data Fig. 5d). However, longer A-rich sequences of 30-nucleotides or random length long polyA phase separate when SERF2 is added (Supplementary Data Fig. 5d, f). We postulate that rG4 structures may have a triggering role in SERF2 phase transition and that the enrichment of rG4 upon stress may attract SERF2-like disordered protein assemblies into stress granules.

### SERF2 and RNA G-quadruplexes form slowly exchanging droplets

SERF proteins have been postulated to be cellular drivers of amyloid formation.^43^ We demonstrate here that SERF2’s interaction with RNA can lead to liquid-liquid phase transition. These observations, along with the fact that the transitions of liquid droplets to more solid gel-like aggregates have been implicated in several neurodegenerative diseases,^73^ motivated us to look at the dynamics of the interactions between SERF2 and RNA. FRAP measurements showed that SERF2 interacts dynamically with bulk HeLa total RNA, showing rapid recovery halftime of 13±2 seconds (Fig. 4h). The half-time recovery of SERF2 in droplets containing long random-length polyA is 34.4 seconds (Supplementary Data Fig. 5f). In contrast, SERF2-rG4 droplets show considerably slower exchange dynamics for both the protein and rG4 components, with halftimes of recovery ranging from ~80 to 620 seconds (Figs. 4c–e, bottom). This indicates that the SERF2-rG4 droplets are less fluid than those that form within stress granules containing long RNA or total RNA in cells.

### SERF2 and RNA droplets facilitate G3BP1 condensation

Having shown that SERF2 is important for stress granule assembly and considering that others have proposed G3BP1 to be a key component in this process,^65^ we asked if the SERF2-RNA interaction affects G3BP1 condensation.^47^ Our findings revealed that in the presence of HeLa cell total RNA, G3BP1 and SERF2 undergo liquid-liquid phase transition (Fig. 4i). As the SERF2 concentration increases, remarkably large condensates up to 4.2 μm in size form that are composed of G3BP1 and total RNA (Fig. 4j). These large condensates were not seen when total RNA was absent (Supplementary Data Fig. 5g). G3BP1 binding to rG4 has been shown to regulate its enrichment and mRNA stability in stress granules,^9,74^ however, whether G-quadruplexes facilitate G3BP1 phase transition is unclear. G3BP1 phase separates both in the presence of total RNA and long random-length polyA RNA that are present in concentrations as low as 25 ng/μL indicating that rG4 structures may not be crucial for G3BP1 phase transition.^74^ We also observed short RNA or rG4 does not promote G3BP1 condensation in a non-crowding environment. Further, the stimulatory effect of SERF2 on G3BP1 condensation in the presence of rG4s was not observed (Supplementary Data Fig. 5h). Given that molecular crowding is present in vivo and the many studies highlighting that crowding promotes phase transition,^75^ we next explored the effect of SERF2 and rG4 on G3BP1 condensation in a minimal crowding environment. A crowding environment with 2.5% PEG8000 does not induce G3BP1 phase transition; however, it does with the introduction of SERF2 and rG4 in the mixture (Fig. 4k and Supplementary Fig. 5i). This suggests rG4 structures can affect G3BP1 phase transition since their addition to non-phase separated mixtures containing SERF2 can cause phase transition. FRAP measurements reveal that G3BP1 recovery in condensate is faster in the presence of SERF2 and SERF2-rG4 mixtures as compared to condensates formed by G3BP1 alone or in the presence of SERF2 and HeLa cell total RNA (Fig. 4l, m). Given the cellular complexity of stress granules, the three-component in vitro model indicates that SERF2 and rG4 enhance G3BP1 dynamics and fluidity. The SERF2 recovery in the G3BP1-rG4 condensates was faster (~20–30 seconds) which correlates to the stress granule dynamics as compared to SERF2-rG4 condensates ~80 to 620 seconds suggesting multivalent weak interactions enhance protein fluidity in multicomponent condensates (Fig. 4c, m). Together, our results suggest that SERF2 promotes G3BP1-RNA condensation in vitro, similar to other stress granule proteins,^6^ and correlate with the observation that SERF2’s depletion results in fewer stress granule foci in vivo (Fig. 2c).

### Human SERF2 is partially disordered

**Fig. 5|:**
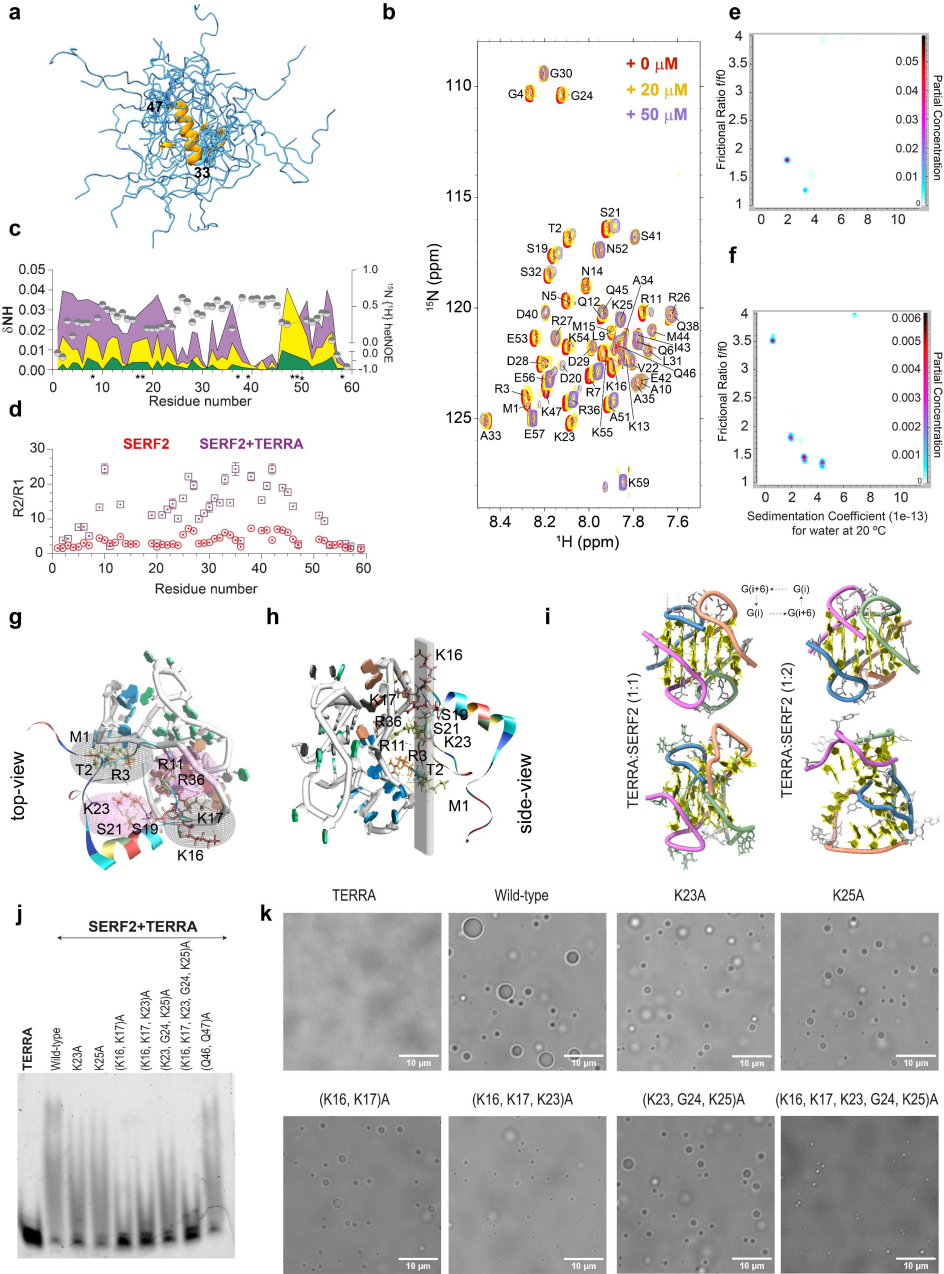
High-resolution NMR structure of SERF2 reveals the disordered and dynamic domain binds rG4 quadruplex. **a,** 20 best NMR ensemble model structures of SERF2 calculated using Cyana and refined using Crystallography and NMR System. The average converged helical structure spanning residues 33–47 in SERF2 is shown in orange. **b,** 15N/1H amide resonance assignment (red spectrum) of 100 μM human SERF2 mixed with 20 μM (yellow) and 50 μM (purple) TERRA rG4 quadruplex at 4 °C. **c,** The 15N/1H chemical shift perturbations (CSPs) were calculated from (**b)** and plotted for each assigned residue in SERF2 with increasing TERRA rG4 quadruplex concentrations. The color codes correlate to the spectrum shown in (**b**) and unassigned peaks are denoted with asterisk. The heteroNOE values of SERF2 in the absence of TERRA rG4 quadruplex were plotted along with the CSPs on the right y-axis to highlight the correlation between SERF2 dynamics and TERRA rG4 interaction. **d,** 15N relaxation rates R2/R1 demonstrating a significant change in 200 μM SERF2 dynamics interacting with 100 μM TERRA rG4 as a function of residues. Protein and RNA samples are dissolved in a buffer containing 8% D_2_O and spectra are recorded at 4 °C on a Bruker 600 MHz spectrometer. **e,f,** 2D analysis plots derived from AUC for 4.7 μM TERRA rG4 quadruplex mixed without (**e**) or with 2-molar excess SERF2 (**f**). The partial concentration shown in color on the right y-axes represents the abundance of individual species in the sample solution. **g,h,** Cartoon shows the top- and side-view of a quadrupole-like (ellipses, **g**) and planar (vertical slab, **h**) interaction in SERF2 and TERRA rG4 quadruplex complex. Residues generating the quadrupole-like interaction and TERRA rG4 quadruplex structure distortion are labeled, and hydrogen bonds are indicated with dashed lines. **i,** 3D structure of TERRA rG4 quadruplex complex with SERF2 before and after 0.5 μs MD simulation shows G-tetrad distortion in TERRA:SERF2 1:1 (center) and 1:2 (right) complex. G-tetrads are indicated by red arrows and each TERRA unit in the tetrameric structure (PDB ID: 2M18) is represented with different colors. A G-quartet in TERRA rG4 quadruplex is formed by guanines in i and i+6 as shown on the top. **j**, EMSA gel-shift assay of 5 μM TERRA rG4 quadruplex mixed with equimolar SERF2 and different lysine to alanine SERF2 mutants in a non-crowding condition (20 mM NaPi, pH 7.4, 100 mM KCl) as indicated in the figure. **k**, Phase transition of SERF2 and its mutants mixed with equimolar TERRA rG4 quadruplexes in a crowding condition (20 mM NaPi, pH 7.4, 100 mM KCl, 10% w/v PEG8000).

Our observation that SERF2 and rG4 interaction can drive phase transition inspired us to attempt to obtain a detailed structural understanding of the ribonucleoprotein condensation process, an important aim that has so far largely eluded researchers. TERRA has already been structurally well characterized,^76^ we thus turned our attention to structurally characterizing SERF2. The narrow (~7.5 – 8.5 ppm) 1H NMR chemical shift dispersion observed even at 4 °C supports the idea that SERF2 is at least partially disordered (Supplementary Data Fig. 6a). The fact that the majority of SERF2’s 15N/1H cross peaks in HSQC spectra are undetectable after a shift to 37° degrees suggests that chemical exchange is occurring in the NMR measurement timescale at this temperature. This may be due to the induction of different conformational states in SERF2, a phenomenon that has been demonstrated to occur with other disordered proteins upon temperature upshift (Supplementary Data Fig. 6a).^77,78^ At 4 °C, SERF2 has a relatively high proportion of helical content, as also measured by CD spectroscopy, but this helicity decreases substantially at more physiological temperatures, consistent with our NMR data (Supplementary Data Fig. 6b). The well-defined nature of the NMR spectra at 4 °C enabled us to assign the backbone (N, Cα, Cβ, NH, CO) NMR chemical shifts for 51 out of 58 non-proline residues in SERF2.^79^ We were able to obtain the torsion angle restraints for the ϕ and ψ angles in the SERF2 polypeptide chain using the TALOS-N program.^80^ Additionally, we obtained 494 NOE restraints for SERF2 from 3D 15N-HSQC-NOESY measurements for SERF2 structure calculation. In combination, these multiple restraints allowed us to build the SERF2 structural ensemble that is shown in Fig. 5a. The N-terminal domain residues 1–32 and C-terminal domain residues 48–59 of SERF2 were observed to be highly dynamic and disordered, whereas residues 33–47 possess a helical structure, as shown in orange in Fig. 5a.

### rG4 binding restrains SERF2 dynamics

A large proportion of RNA interaction motifs in proteins are disordered,^81^ but the role that this disorder plays in RNA-protein interactions is not yet clear. Fortunately, in our case, the NMR methods available for the SERF2 protein and the RNAs it interacts with have enabled us to examine in depth 1) the specific interactions that occur between SERF2 and rG4, 2) the role that disorder plays in these interactions, and 3) how these interactions impact the structural dynamics of both binding partners. To map the protein binding sites, we studied its interaction with the TERRA rG4. We first monitored the 15N/1H chemical shift perturbations that occur in SERF2 with increasing TERRA concentrations (Figs. 5b and c). TERRA binding induces major chemical shift perturbations in both the N-terminal (3–21) and C-terminal (51–56) residues of SERF2. At the lowest TERRA rG4 concentration of 5 μM against 100 μM ^15^N-labeled SERF2 (Fig. 5c, green), residues R7, R11, K16, K23, R27, Q46, Q47 and K55 showed a substantial chemical shift perturbation. SERF2 at the lowest concentration of 10 μM titrated against 100 μM natural abundance TERRA induced chemical shift perturbations for G5, G9, and G10 imino protons of TERRA suggestive of the direct or indirect involvement of these guanines in interaction with the SERF2 protein (Supplementary Data Fig. 6c). Saturation transfer difference NMR measurements further revealed magnetization transfer only occurs from TERRA guanine imino protons when mixed with SERF2 (Supplementary Data Fig. 6d, e). Similarly, the saturation of the A33 amide proton in SERF2 that does not overlap with any RNA signals (Supplementary Data Fig. 6f, g) also shows magnetization transfer to TERRA guanine imino protons. Together, these data suggest that SERF2 is spatially oriented close to the TERRA G-quartet core upon interaction. To examine if the disordered and evolutionary conserved N-terminal domain in SERF2 which shows a strong chemical shift perturbation (Fig. 5c) dominates over the C-terminal domain in rG4 binding, we determined the binding affinity of individual domains in SERF2. The N-terminal with 32 residues of SERF2 on its own, binds TERRA rG4 quadruplex ~10-fold weaker than the binding of full-length SERF2. The C-terminal domain of SERF2 (residues 31–59), in isolation, showed no detectable binding (Supplementary Data Fig. 6h). Together, these results suggest that the N-terminal domain is more important for rG4 recognition, though the N- and C-termini appear to coordinate with one another to form a tight SERF2-TERRA complex.

To test whether SERF2 dynamics change upon rG4 binding, we compared the NMR relaxation data of SERF2 in the absence and presence of TERRA rG4 to determine residue level resolution dynamic information. When SERF2 dynamics in isolation (Figure 5c, gray spheres) is plotted against residues that show a stronger chemical shift perturbation upon rG4 interaction, we observed a strong correlation between SERF2 dynamics measured by a low heteronuclear NOE value and its rG4 binding interface. Specifically, the unstructured regions, spanning residues 3–24 and 48–56, are highly dynamic in SERF2, with average heteronuclear NOEs < 0.3 and they also show the highest chemical shift perturbations and change in peak signal height upon binding to TERRA rG4 (Fig. 5c and Supplementary Data Fig. 6i). These results suggest that the flexible regions in SERF2 are involved in rG4 binding. Since both the structurally disordered, dynamic regions are directly involved in the interaction with the rG4, it seems possible that disorder is a prerequisite for rG4 recognition. The decreased SERF2 residue signal intensities at increased TERRA rG4 concentration are inversely correlated to the spin-spin (T2) relaxation rates. This may be the result of several factors such as conformational exchange upon TERRA binding, an increase in complex size leading to decreases in correlation times, or amide proton exchange with bulk solvent. The overall increase in backbone R2/R1 relaxation rate of individual SERF2 residues upon TERRA rG4 binding indicates SERF2 dynamics is relatively constrained in the complex with rG4 as compared to an unbound state (Fig. 5d).

### Examining the initial interactions that drive phase transitions

Multivalent interactions are critical for driving in vitro phase transitions, particularly in systems involving disordered proteins, which are known to facilitate liquid-liquid phase transitions.^82^ However, the specific molecular species and mechanisms that nucleate phase transitions within ribonucleoprotein condensates remain poorly understood. Given SERF2’s involvement in RNA-driven phase transitions and its favorable biophysical properties, we aimed to leverage this system to gain detailed structural insights into the early interactions that occur during the initiation of phase separation.

Size-exclusion chromatography analysis demonstrated the existence of at least three different-sized species of TERRA rG4 that absorb at 260 nm after it is mixed with SERF2. The absorption detected at 260 nm comes solely from TERRA, as SERF2 is tryptophan- and tyrosine-free and thus conveniently does not absorb at 260 nm. The absorbing species are of sizes consistent with being unbound TERRA rG4 structures, which eluted at ~15 kDa, the sole peak that is present in the absence of SERF2, a 1:1 protein-TERRA complex, that eluted at ~30 kDa, and one or more multimeric species that elute as a peak >30 kDa (Supplementary Data Fig. 6j). To determine the protein-TERRA rG4 stoichiometry in these multimeric species, we performed analytical ultracentrifugation. In isolation, the TERRA rG4 shows two major polymorphic structures with sedimentation coefficients of 1.95 and 3.33, suggestive of globular, folded structures (Fig. 5e). In a SERF2 and TERRA mixture, three additional species were observed with higher sedimentation coefficient values and predicted sizes of ~ 12.4, 17.4, and 27.1 kDa for sedimentation values 1.95, 3.00, and 4.38, respectively, suggestive of 1:0, 1:1, and 1:2 TERRA: SERF2 complexes. The higher molecular weight TERRA-SERF2 complexes were observed to be globular with a lower frictional ratio, whereas the smaller complex constitutes elongated or unfolded TERRA species (Fig. 5f). This size-distribution analysis indicates that SERF2 forms multimeric species in complex with rG4 and supports their tendency to phase separate.

To examine the structural dynamics and stability of the SERF2-rG4 complex structure, we built a 1:1 or 1:2 complex structure using binding constraints obtained from NMR experiments (Fig. 5c and Supplementary Data Fig. 6c–f). The modeling platform HADDOCK^83^ was used to build the complex structures of the 1:1 and 1:2 (TERRA:SERF2) as supported by analytical ultracentrifugation. A set of ambiguous binding site residues obtained from NMR titration and saturation transfer difference measurements were input to the HADDOCK program (see methods). The lowest energy complex model structures were subjected to microseconds scale all-atom molecular dynamic simulation to obtain some understanding of the complex structural changes and dynamics that accompany SERF2 and rG4 interaction in the early stages and during liquid-liquid phase transitions. The structure obtained after a 0.5 μs MD simulation retains N-terminal contacts in 1:1 TERRA:SERF2 complex (Fig. 5g) but does not show any C-terminal residue contacts. On the contrary, in the 1:2 TERRA:SERF2 complex, the C-terminal contacts are gained in addition to the N-terminus contacts (Supplementary Data Fig. 7a) that correlate with the NMR observations. These interactions are important for providing multivalent contacts in SERF2 that are important for the formation of the oligomeric species and possibly the liquid-liquid phase transitions to occur.

Structural analysis of SERF2-rG4 complex showed a hydrophilic core in the complex structure formed by SERF2 residues located in regions that are dynamic in monomeric SERF2 (Fig. 5g, h). This hydrophilic core constrains SERF2 dynamics by generating a planar contact surface that interacts with the planar G-quartets through a quadrupolar-like contact architecture. Several N-terminally located charged residues (R3, R11, K16, K17, and K23) showed a high propensity to form hydrogen bonds with the TERRA rG4 quadruplex G1, G3, G7, G8, and G9 nucleotides located in the bottom two G-quartets that are formed by the interaction of two TERRA molecules (Fig. 5g). The planar and quadrupolar-like contact surface between SERF2 and TERRA is formed by a polar surface comprised of charged residues (Fig. 5g, 5h). The contact surface formed by the first three N-terminal SERF2 residues M1, T2, and R3 faces opposite to the binding surface formed by two charged residues K16 and K17. Similarly, the charged residues R11 and R36 in one contact surface face opposite the other polar surface formed by residues S19, S21, and K23 (Fig. 5g). The guanines in the two-dimeric TERRA rG4 quadruplex structure (PDB ID: 2M18)^71^ are bonded in a nucleotide number ‘i’ to ‘i+6’ pattern to form 5 neatly stacked G-quartets (Fig. 5i). Interestingly, our all-atom simulation revealed SERF2 binding in the 1:2 complex distorts two G-quartets and affects the TERRA rG4 quadruplex structural integrity (Fig. 5i). The G9-G3 Hoogsteen bond in one interface is disrupted by SERF2 binding residues R11 and R3. On another binding interface, SERF2 residues S19-S21-K23 and M1-T2 interaction solvent exposed the buried TERRA rG4 nucleotide ‘U4’ that stabilizes the rG4 quadruplex structure through hydrogen bonds with G2 and G3 (Fig. 5g, Supplementary Data Fig. 7b). K16-K17 residues of SERF2 by forming hydrogen bonds with G7 and G8 of TERRA interrupts the G7-G1 and G7-G8 Hoogsteen base pairing.

The hydrophilic core in the 2:1 SERF2:TERRA complex is bigger than that present in the 1:1 complex. This is due to the incorporation of several charged C-terminal residues including R36, K50, K54, and K55 from SERF2 that form hydrogen bonds with TERRA (Supplementary Data Fig. 7c). Hydrogen bond profiling versus atomic simulation time-scale showed that SERF2 tends to interact with uracil nucleotides in the 1:1 TERRA:SERF2 complex and then shifts towards a guanine nucleotide interaction in the 1:2 complex (Supplementary Data Fig. 7d). Interaction of guanine nucleotides located in quartets with helicases such as Pif1 and DHX36 are shown to be important for rG4 quadruplex unfolding.^84,85^ To see if we could obtain experimental evidence for this structural distortion predicted by our MD simulations, we studied the TERRA rG4 quadruplex secondary structure in the presence of SERF2 by CD and NMR spectroscopy. The parallel structure content of TERRA, as measured by CD, gradually decreased with increasing SERF2 concentration and NMR shows the disappearance of imino proton signals upon an increasing concentration of SERF2 (Supplementary Data Fig. 6c, 7e). Though these findings correlate with the atomistic MD simulation results; the MD simulation appears to overrepresent the structural distortion. We also observed TERRA rG4 structure stability with slight distortion (Supplementary Data Fig. 7f, g) simulated in isolation with sufficient K^+^ ions (150 mM KCl). Therefore, we explain the stronger structural distortion observed in our MD simulation as compared to our CD results could be due to the partition of K^+^ ion into the tetrad-quartets similar to what has previously been observed^86^ and K^+^ ion dissociation upon SERF2 binding. The high-resolution structural details obtained from MD simulations that show the TERRA rG4 binding to multiple charged interfaces at N- and C-terminus in SERF2 molecule may reflect the interactions expected in the early stages of liquid-liquid phase separation, a process known to form through multivalent interactions.^2^ The pattern of charged residues in disordered proteins and the charge interactions are demonstrated to play a crucial role in the liquid-liquid phase transition.^87^ The distortion of nucleic acid structures, such as unwinding or bending, could enhance these interactions by exposing charged regions, shielding RNA-RNA interactions,^88^ and promoting multivalent intermolecular interactions essential for phase separation. Together, the simulation results suggest that charge interactions and nucleic acid conformational changes help stabilize the dynamic condensates and facilitate their selective recruitment of specific proteins and nucleic acids during phase transition.

### Mutational study reveals multivalent interaction drives SERF2 phase transition

Our MD simulation and NMR results indicate residues and interfaces in SERF2 that appear to be directly involved in rG4 binding. To experimentally test how important these residues and interfaces are for rG4 binding, we next perform a series of mutagenesis studies of conserved lysine residues that included single point mutations, and multiple mutations designed to eliminate either a single-binding interface (K16, K17) or multiple-binding interfaces (S21-R27) (Fig. 5h, j). A gel-shift binding assay shows that independent mutation of the evolutionarily conserved lysine residues K23 or K25 to alanine has a minimal effect on TERRA rG4 binding. In contrast, the disruption of both residues K16 and K17, which are predicted to form one binding interface, reduces SERF2 binding to the TERRA rG4 (Fig. 5j). Interestingly, this same mutation in SERF2 also fails to bind its amyloid substrate.^89^ Similarly, disruption of the second binding interface by alanine substitutions of residues K23, G24, and K25 weakens SERF2 and TERRA rG4 interaction. Mutation of all conserved lysine residues in the two major binding interfaces (residues K16, K17, K23, G24 and K25) significantly reduces TERRA rG4 binding (Fig. 5j). In contrast, mutation of two C-terminal residues Q46 and Q47 that show a higher chemical shift perturbation in the NMR titration experiment shows no effect on TERRA rG4 binding suggesting the C-terminal domain is not crucial for rG4 binding. We further explore the effect of the multi-interface interactions in SERF2 with TERRA rG4s in phase separation under crowding conditions. Differential interference contrast microscopy imaging shows point lysine mutants K23 or K25 do not affect SERF2-TERRA phase separation. Surprisingly, alanine substitutions of the individual interface residues K16-K17 or K23-G24-K25 which substantially affect TERRA binding in a non-crowding condition (Fig. 5j), show phase separation in a crowding condition (Fig. 5k). However, alanine mutations that affect both of the two binding interfaces i.e. K16-K17-K23 or K16-K17-K23-G24-K25 do affect SERF2 phase separation (Fig. 5k). These results suggest SERF2 engages multiple binding interfaces to mediate multivalent interactions that drive their phase separation.

### High-resolution structural insights into ribonucleoprotein condensate formation

Biomolecular condensation is emerging as an important factor in many cellular functions and human diseases;^90^ however, we currently have a limited high-resolution understanding of this process and so far only for protein-only condensates.^2,91–93^ Our NMR data obtained under non-crowding conditions, in combination with all-atom MD simulations, have revealed many details concerning how SERF2 and rG4 interact and how their structural ensembles alter upon interaction. However, these NMR experiments are limited to soluble protein-RNA complexes, as the addition of molecular crowding agents to these mixtures rapidly results in the formation of condensates and NMR signal broadening. Therefore, to study these ribonucleoprotein droplets at atomic resolution, we introduced crowding into our large-scale all-atom simulations that mimic the molecular crowding present in vivo and in our in vitro experiments. We reasoned that examining how the RNA and protein components interact in these simulations may allow us to visualize phase separation at high resolution. In particular, we examined if the structural interactions observed under non-crowded conditions are recapitulated in a crowded condition. This reasoning is predicated on the assumption that the type of interactions between protein and RNA do not significantly change upon the addition of crowding agents, and that condensation is mainly due to an increase in the valency of interactions driven by crowding. Our assumption also accommodates the stickers and spacers model framework in phase separation where the sticker residues generate multivalent interactions, and the spacer residues provide structural plasticity.^94^ In support of our assumption are experimental observations indicating that the proteins and RNAs that interact within condensates also interact in the dilute phase making condensates macroscopically visible manifestations of weak and transient interactions that take place normally in the cell.^95^

**Fig. 6|:**
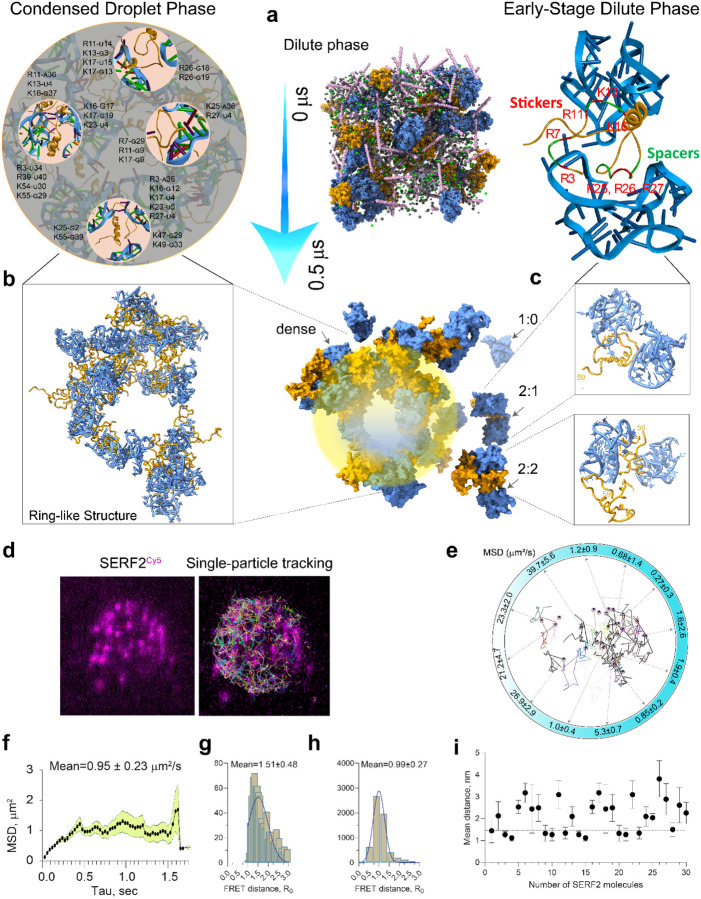
Integrated single-molecule fluorescence microscopy and MD simulation approach to study high-resolution structure of SERF2 – TERRA rG4 quadruplex phase separated condensate. **a,** A cubic all-atom MD simulation box encapsulating randomly distributed 30 molecules SERF2 (orange), 30 molecules of TERRA rG4 quadruplex (blue), 10% PEG (pink), Cl^−^ (green), and K^+^ (grey). The downward arrow on top indicates the transition of a dilute phase low-ordered structure to a more droplet-like condensed phase structure in 0.5 μs large-scale MD simulation. **b,c,** Surface representation of the structure of SERF2-TERRA rG4 ring-shaped condensed droplet-like large-structure **(b),** and lower-ordered oligomers **(c)** obtained at time 0.5 μs MD simulation. The enlarged all-atom cartoon structures of the condensed droplet-phase and the lower-ordered 1:2 SERF2:TERRA rG4 dilute-phase oligomer are shown on the top. The three distinct contact sites in SERF2 in the lower-ordered complex structure (**c,** top) is shown in ball-stick format and the TERRA rG4 interacting residues are labeled. The high-resolution images in (**b**, top) show a representative interaction network that involves the three critical binding sites in SERF2 located in the disordered N-terminus. The SERF2 interacting residues are represented with an uppercase letter and TERRA rG4 quadruplex nucleotide in a smaller-case letter (e.g. R11-U14 denotes Arg3 and uracil 14 in SERF2 and TERRA rG4 quadruplex, respectively). **d**, (left) Single-molecule fluorescence microscopy shows a single droplet of 50 μM SERF2 (spiked with picomolar Cy5 labeled SERF2) mixed with equimolar TERRA rG4 quadruplexes. The corresponding single-particle tracking image of SERF2 molecules within the single droplet generated using ImageJ is shown on the right. **e**, Single-molecule tracking image constructed using the 0.5 μs all-atom MD trajectory in the SERF2-TERRA all-atom MD simulation system. Each sphere represents the center of mass of individual SERF2 molecules, and their trajectories were retrieved at every 20 nanoseconds interval from the 0.5 μs MD simulation to construct the tracks using ImageJ. The mean square displacement (MSD) of a set of representative SERF2 molecules in the lower-ordered dilute and droplet-like condensed phase are indicated by arrows. **f,** MSD plot of SERF2 (average MSD 0.95±0.23 μm^2^/s) obtained from the single-particle tracking fluorescence microscopy experiment. **g,h**, FRET distance histogram between donor and acceptor was determined based on the FRET efficiency in SERF2 (T2C and A51C) and TERRA rG4 phase-separated droplets containing picomolar Cy3-Cy5 labeled SERF2 in dilute-phase **(g)** and condensed-phase **(h)** samples separated after centrifugation (see methods). **i**, Mean distance between T2 and A51 in SERF2 was computed from 0.5 μs MD simulation in a system containing 30 SERF2 molecules and 30 TERRA rG4 molecules. The dashed line indicates molecules with a mean distance of 1.5 nm.

To explore the structural paradigm of protein and RNA condensation, we thus wanted to examine if our high-resolution observations concerning SERF2 and TERRA rG4 could be extended beyond the solution phase. By employing large-scale all-atom MD simulations, we aim to explore the structural interactions between SERF2 and TERRA rG4 in an environment mimicking an in vitro or in vivo crowding condition where phase separation occurs. We started our all-atom simulation using a collection of ~0.3 million atoms including 30 molecules of SERF2 and 30 of TERRA rG4, 100 mM KCl aqueous solution in a random loose arrangement that also included 10% v/v PEG to favor condensation (Fig. 6a). The final MD snapshot retrieved at 0.5 μs MD simulation presented some unbound TERRA molecules, small SERF2-TERRA 1:1 and 1:2 oligomers, but was dominated by a densely packed higher-ordered ring-like structure of SERF2 and TERRA molecules surrounding a hydrated core (Fig. 6b, 6c). The interaction network analysis of the lower-ordered 2:1 SERF2-TERRA complex showed a four-site binding involving N-terminal residues M1-R3-R7, R11, K16-K18, and K25-R26-R27 (Fig. 6c) that resembles the SERF2 binding to TERRA observed in our non-crowding MD simulation system (Fig. 5h). This observation supports our assumption that the MD simulation results obtained in a non-crowded system correlate to the early stages of liquid-liquid phase transition. Interaction analysis of the 2:2 SERF2-TERRA oligomer obtained at 0.5 μs of MD also showed the major interacting residues in SERF2 are centered at the N-terminus with additional contacts from the C-terminus, enabling a multivalent interactions network known to influence phase separation (Supplementary Data Fig. 7h).

In examining the structural interaction network within the higher-ordered ring-like structure, we found that the densely packed region consisted of a series of multivalent interactions that bring multiple TERRA rG4 molecules into interaction with one SERF2 protein and vice versa. As illustrated in Fig. 6b (top), a single SERF2 molecule showed interaction with three TERRA rG4 molecules through a large, charged interface involving residues R3, K16, K17, K23, and R27 that coordinate one TERRA rG4, and two smaller interfaces interacting two other TERRA rG4 molecules through residues K25-K55 and K47-K49. Similarly, two SERF2 molecules are shown to bind two TERRA rG4s in a fashion where one tightly engages its N- and C-terminus, while the other shows a weak contact map through region R11-K16 and exposes its N- and C-terminus hinting at likely recruitment of additional TERRA partners, a pattern observed for the dilute phase 2:2 complex (Supplementary Data Fig. 7h). Interaction network analysis (as described in the methods) showed the charged residues are predominantly engaged in TERRA interaction and their bonding occupancy and multivalency increases as the system size increases (Supplementary Data Fig. 7i). In the simulated condensed state, the majority of the SERF2 molecules were seen to bind two TERRA rG4s generating a ring-shaped polymeric structure (Fig. 6b, Supplementary Data Fig. 7j and Supporting Video SV1). This distinctive ring-shaped structure was formed at earlier simulation times and a similar morphology was identified in a smaller system comprising only six SERF2 and TERRA molecules within 1 μs (Supplementary Data Fig. 7k).

### Single-molecule tracking of biomolecular condensates

To validate our MD simulations, we attempted to directly compare the MD simulation results with experimental data. A crucial feature of biomolecular condensates is the residence time of polymers that correlates to their molecular diffusion within a microenvironment through intra- and intermolecular interactions. We hypothesize that under a similar condition, the *in silico* computed diffusion coefficients of SERF2 and rG4 molecules in condensates should closely resemble an experimentally derived diffusion constant as has been previously demonstrated for protein-only condensates.^2,93^

We thus carried out single-particle tracking to experimentally determine the mean-square displacement of SERF2 molecules within the condensates (Fig. 6d) and compared it to the mean-square displacement of SERF2 molecules in the higher-ordered states of the MD trajectory (Fig. 6e). The diffusion constant we calculated for SERF2 molecules present in the dimer and small oligomer portions of the MD simulation, which we consider to be the dilute phase, was, as expected rather high, at ~20 to 40 μm^2^/s (Fig. 6e). The average mean-square displacement was 4.91±0.96 μm^2^/s, and that of individual SERF2 molecules in the higher-ordered structure seen in the MD simulation range was much lower ranging from ~0.3 to 5 μm^2^/s (Fig. 6e). These values are comparable to the experimentally determined average meansquare displacement of 0.95 ± 0.23 μm^2^/s obtained using single-molecule tracking of SERF2 present within the condensates (Fig. 6f). The SERF2 molecules outside of these condensates in the diffuse phase are moving too fast to accurately measure. The rates of SERF2 diffusion within the condensates are also similar to diffusion rates observed for DNA binding proteins in the presence of DNA.^96^ For instance, the diffusion rates measured for proteins found within nucleoprotein or reconstituted postsynaptic density condensates range from ~0.1 to 0.6 μm^2^/s,^96,97^ whereas in protein-only condensates a broad range of diffusion rates (~1 to 100 μm^2^/s) have been reported.^2,93^

The slow diffusion of SERF2 bound to the TERRA rG4 within the condensates is likely due to the strong electrostatic interactions we observed in our all-atom MD simulation. That strong electrostatic interactions occur is experimentally supported by the observation that SERF2-TERRA rG4 droplets are stable at non-physiological salt concentrations (Supplementary Data Fig. 8a). These modeled interactions act through multiple binding sites consisting of charged lysine and arginine residues as has been observed for other nucleic acid binding proteins.^96^ The slower fusion rate of proteins that are present in ribonucleoprotein droplets than are observed in protein-only droplets indicates a more viscous and rigid structural organization within ribonucleoprotein droplets. The fluid nature of SERF2 molecules within the ribonucleoprotein condensates and their translational diffusion was next tested. Single-droplet fusion using a dual-trap optical tweezer showed the fusion time for SERF2 and TERRA rG4 droplets range from ~2 to 4 seconds (Supplementary Data Fig. 8b), which is one to two orders of magnitude slower than that observed for protein-only condensates.^2,93^ This is further reflected in the translational diffusion rate of SERF2 measured using FRAP which showed a slow half-time recovery of ~2 minutes (Supplementary Data Fig. 8c), in contrast, the recovery rates observed for protein-only condensates occur on the second timescale.^2,93^

To further understand the dynamics and spatiotemporal organization of SERF2, we calculated the end-to-end distance between the N- (T2C) and C-terminal (A51C) using static FRET measurements in diluted and condensed phase samples. The mean distance between T2C and A51C in SERF2 molecules in the dilute phase was measured to be 1.51±0.5 nm (Fig. 6g) suggesting the N- and C-termini in SERF2 are adjacent. This is consistent with our NMR observations of the SERF2 structure that shows a strong paramagnetic relaxation effect employed by a paramagnetic tag at T2C on A51.^79^ SERF2 molecules are more compact in the condensed phase with a mean diameter of 0.99 ± 0.27 nm (Fig. 6h). We note that considering the experimental limitations and assuming a random orientation of SERF2 within the condensate, an accurate distance estimation is not possible between a donor and acceptor. The structural orientation of the SERF2 molecule was next assessed by computing the mean distance between residue T2 and A51. A mean distance of 2.09±0.2 nm was obtained for SERF2 molecules (Supplementary Data Fig. 8d), a value slightly higher than the average distance as measured by FRET. Single-molecule distance analysis however presented several SERF2 molecules having an end-to-end distance within a range of ~1.1 to 1.3 nm (Fig. 6i) that falls within a range of mean distances measured for dilute and condensed phase SERF2 molecules by FRET. We conclude that SERF2 retains a compact structure within the ribonucleoprotein condensates.

## Discussion

There is a large range of liquid-liquid compartments in the cell. Though the precise function of this form of compartmentalization is unclear, one common feature is that they act to concentrate the molecules present within them. Perhaps the most well-characterized of these compartments is the nucleolus which is important for an efficient ribosome assembly.^46^ Another well-characterized compartment is the stress granule which may be involved in RNA or protein storage during stress.^98^ Many of the proteins within liquid-liquid phase compartments contain elements of disorder and understanding how they function and interact with other molecules is limited. This is due to a lack of experimentally determined conformational ensembles for disordered proteins making it difficult to study their interaction with RNA species. We have a broad overview of factors affecting liquid-liquid phase transitions including the importance of multivalency and disorder. However, the lack of detailed structural information leaves both the protein and RNA components of these membrane-less compartments often diagramed as vague spaghetti-like lines. Our replacing these vague lines, with the structural and biophysical details diagrammed in Figures 5 and 6, helps us understand the process of liquid-liquid phase separation and the interactions that take place within these recently recognized compartments.

Here we show that SERF2, initially isolated for its ability to accelerate amyloid formation,^40^ is a component of the nucleolus and stress granules and appears to be important for stress granule formation. The formation of stress granules is a complex process, with several proteins that appear to influence their assembly, stability, or both,^4,6,7,49,65^ with an emerging consensus that the core proteins G3BP1 and G3BP2 are particularly important for the formation of stress granules,^6,99^ The overproduction of SERF2 like G3BP1 and G3BP2, also triggers the appearance of stress granules in the absence of stress and their depletion reduces the granule distribution.^4,7^ Two high-throughput screening approaches, supported by a series of biophysical experiments show that SERF2 has a strong tendency to bind to rG4 structures. rG4s have been implicated in a variety of functions such as translational repression and transcriptional termination,^39^ mRNA processing, mRNA polyadenylation and splicing, telomere maintenance, and RNA translocation.^39,61,100–103^ However, their mechanisms of action remain unclear.^43,104^ In findings likely relevant to ours, it has recently been found that stress enhances the number of rG4 present.^28^ rG4 are abundant in stress granules, and stress promotes rG4 folding.^28^ We hypothesize that rG4 may facilitate the formation of stress granules, a cellular compartment that may be involved in maintaining proteins in a soluble form which is consistent with recent observations for rG4 being anti-aggregation agents.^26,105,106^

A handful of RNA-binding proteins, such as FMRP, nucleolin, CNBP, eIF4A, hnRNPA1, and DHX36, have been shown to bind rG4 and modulate their folding.^30–34,36^ SERF2 binds to known rG4 structures with sub-micromolar binding affinities similar to the affinities of other rG4 binding proteins including cold-inducible RNA-binding protein,^107^ FUS,^108^ FMRP,^109^ as well as G-quadruplex binding small molecules such as pyridostatin, NMM, and BRACO-19.^110^ A common feature of these binding proteins is the engagement of an intrinsically disordered domain in the binding, however, a lack of detailed structural and residue-level dynamic information in these complex systems has limited our understanding of the role that disorder plays in these systems. Upon interaction of SERF2 with rG4, a planar G-quartet RNA-protein interaction forms through a quadrupole-like interaction. The SERF2 protein dynamics are constrained upon rG4 binding, and the rG4 structure becomes distorted (Fig. 5). The conformational adaptability of SERF2 enables it to interact with the TERRA rG4 structure which induces destabilization in the TERRA rG4 structure indicative of SERF2 being involved in rG4 unfolding. Planar interactions of small molecules with G-quadruplexes have been used in structure-based small-molecule design for both G4 stabilization^111^ and destabilization.^112^ Further, proteins have shown such interactions to be able to regulate their cellular functions, for instance, yeast Rap1 interacts with G4 using planar G-quartets to stabilize the complex.^113^ In contrast, the DHX36 helicase unfolds G4^85^ by forming a flat non-polar surface on the G-quartet, in a way similar to that observed for SERF2.

If rG4 structures thermodynamically stabilize the SERF2 protein, they may affect the levels of SERF2 in vivo and thus affect its ability to partition to liquid-liquid compartments such as the nucleolus or stress granules.^114^ As rG4 folding elevates the local charge density, it may be possible that stress that triggers rG4 folding not only attracts charged misfolded proteins but also modulates the stress granule dynamics. SERF2 can form liquid-like droplets in vitro and facilitates the in vivo assembly or stability of the stress granule – a liquid-liquid phase transition compartment. Notably, unlike droplets that have undergone a liquid-to-gel transition, or aggregates characterized by extremely slow or no measurable exchange,^115^ those formed between SERF2 and rG4 are still reversible, consistent with the dissipation of stress granules seen upon removal of stress conditions.^7^

Ribonucleoprotein condensates are emerging key cellular players in regulating many biological functions including in human diseases.^82,88,116–118^ However, the molecular architecture of biomolecular condensates is difficult to explore experimentally due in part to the heterogenous and transient nature of the interactions present within these condensates. Many laboratories have instead turned to simplistic mathematical models in an attempt to visualize these condensates at atomic resolution.^91–93^ The stickers-and-spacers model^119^ for instance, uses sticker residues to drive multivalent interactions in disordered proteins while the spacer residues contribute to structural plasticity. However, it is not yet clear if this model is compatible with observations that collective residue interactions act to drive phase separation.^93^ Our studies on SERF2 and the TERRA rG4 provide a high-resolution structural framework for understanding protein-RNA condensates and how multiple transient electrostatic interactions can drive oligomer formation. Our studies bring experimental detail to help refine and validate or invalidate various mathematical models for condensate formation. Our evidence that SERF2 is important for the assembly of stress granules, a major type of liquid-liquid condensate present in vivo, provides physiological relevance to our findings. Unexpectantly, we frequently observed ring-like structures that bring multiple SERF2 and TERRA rG4 molecules together through multivalent interactions in our all-atom MD simulations. These rings represent an attractive mode of interaction that could act to maintain an appropriate degree of exchangeability of the condensate components while avoiding higher-order interactions that could form insoluble aggregates with non-exchangeable components.

Given SERF’s action in speeding protein aggregation and our observations that SERF2 is important for stress granule formation and can engage in liquid-liquid phase separation, it is tempting to consider possible links between these processes. Stress introduced by overexpressing amyloidogenic proteins can lead to persistent stress granules leading to protein aggregation.^120^ Within the high-concentration environment of the stress granule, fibrillization of amyloid may be accelerated by SERF2. G4s have been linked to protein folding in several somewhat disparate ways. G4s have previously been shown to be very potent in inhibiting protein aggregation^106^ and can directly accelerate the folding of model proteins.^121^ On the other hand, it has also been observed that G4 binding to the SERF family protein Znf706 can inhibit Znf706’s ability to accelerate amyloid formation.^44^ We wonder if SERF2 family members interacting with G4 may affect the protein folding activities of both G4 and SERF family members. An appropriate balance of the anti-aggregation activity of G4 and the pro-aggregation properties of SERF2 may be necessary for maintaining liquid-like droplets in a dynamic and reversible state and preventing solidification reactions that have an irreversible effect on protein structure and function.

Though the current work provided structural insights into SERF2 binding to TERRA rG4 and how it distorts the rG4 structure, future work will be necessary to probe if it interacts similarly or differently with in vivo rG4 binding partners, in stress granules or elsewhere. Determining if the loose ring-shaped structures we have observed are a common feature within condensates likely awaits the development of other protein-RNA pairs that can be analyzed in the same detail as our SERF2-rG4 TERRA pair. A better understanding of the structure-function relationships present within quadruplexes will aid us in understanding rG4-linked biological functions such as gene regulation and stress granule formation.

## Methods

### Cell Culture, Treatment, and Transfection

All cells were cultured in Dulbecco’s Modified Eagle Medium (Fisher Scientific, 11-995-073) supplemented with 10% heat-inactivated fetal bovine serum (Sigma, F4135) and 1X Penicillin-Streptomycin-Glutamine (Fisher Scientific, 10-378-016). Stress treatments were performed on cells with vehicles alone or with different stress inducers. Cells were then treated with a final concentration of 0.4M sorbitol or 0.5 mM sodium arsenite to induce osmotic or oxidative stress, respectively. For ER stress, the cells were additionally treated with a final concentration of 2 mM dithiothreitol for 30 minutes in a culture medium at 37°C. Cells were treated with 10 μM of the proteasome inhibitor MG132 (Cas No- 133407-82-6, Sigma) diluted in culture medium and incubated for 30 minutes at 37°C to inhibit protease function. For mitochondrial stress, cells were treated with 75 mM NaN_3_ for 30 minutes at 37°C. For heat shock, cells were incubated at 43°C for 1 hour. For plasmid transfection, cells were transiently transfected using Lipofectamine^™^ LTX and PLUS^™^ reagent (Fisher Scientific, 15338030) according to the manufacturer’s instructions. For knockdown, 13 μM of control (Horizon Discovery, D-001206-13-20) or SERF2 (Horizon Discovery, M-016317–01-0010) siRNAs were transfected using RNAiMAX reagent (Thermo Fisher, 13778150). After 48 h of incubation, the transfected cells were harvested for western blot analysis and RT-qPCR.

### Immunofluorescence

Cells were grown on coverslips with the conditions described above and then fixed with 4% paraformaldehyde (Electron Microscopy Sciences, 157–8-100) in 1X DPBS (Fisher Scientific, 14–190-144) at room temperature for 10 minutes. The fixed cells were washed three times with 1X DPBS and then permeabilized with 0.1 % Triton X-100 at room temperature for 30 minutes. The permeabilized cells were next blocked at room temperature for 1 hour using UltraCruz^™^ blocking reagent (Santa Cruz, sc-516214), followed by incubation with primary antibody solutions for 1 hour at room temperature. Primary antibody solutions were prepared in UltraCruz^™^ blocking reagent using the following dilution factors: 1:200 rabbit anti-SERF2 (Proteintech, 11691–1-AP), 1:1000 mouse anti-G3BP1 (BD Biosciences, 611127), 1:1000 mouse anti-Fibrillarin (Boster Bio, M03178–3), 1:100 mouse anti-Nopp140 (Santa Cruz, sc-374033), 1:100 mouse anti-USP10 (Santa Cruz, sc-365828), 1:200 mouse anti-FUS (Thermo Fisher, 50–554-337), 1:500 mouse anti-BG4 (Absolute antibody, Ab00174–1.1), 1:100 mouse anti-TIA1 (Santa Cruz, sc-398372), and 1:100 mouse anti-eIF2α (Santa Cruz, sc-133132). The cells were then washed three times with 1X DPBS and incubated in secondary antibody solutions in the dark at room temperature for 1 hour. Secondary antibody solutions were prepared in a blocking reagent using a 1:1000 dilution, goat-anti-rabbit secondary Alexa Fluor Plus 488 (Thermo Scientific, A32731), and goat-anti-mouse secondary Alexa Fluor 647 (Thermo Scientific, A21235), goat-anti-mouse secondary Alexa Fluor 488 (Invitrogen, A11001). After three washes with 1X DPBS, the cells were stained with 0.25 μg/mL DAPI (Thermo Scientific, D1306) in 1X DPBS for 3 minutes. The cells were washed three more times with 1X DPBS, air dried, and mounted with ProLongGlod^™^ antifade reagent (Cell Signaling, 9071S) on glass slides. After 24 hours of mounting, the slides were sealed with nail polish and imaged using a Leica SP8 confocal or Thunder^™^ microscope.

### Oligonucleotide synthesis

High-performance liquid chromatography (HPLC) purified unlabeled and 6-FAM fluorescent labeled RNA nucleotides were either purchased from Integrated DNA Technology (IDT) or synthesized at Slovenia NMR Center using previously described methods.^122,123^ Homo-ribopolynucleotides (polyA, polyU, polyC, and polyG) were purchased from Sigma. All the oligonucleotides were suspended in nuclease-free water or buffer prepared using nuclease-free water. All oligonucleotides were desalted using a 3 kD cutoff filter (Amicon ^®^Ultra 0.5 mL) by treating with 20 mM Tris-HCl, pH >11, heated at 95 °C for 3 minutes following 10-time buffer exchange in 20 mM Tris-HCl, pH 7.4. The oligonucleotide concentration was next measured using the extinction coefficients obtained using the IDT OligoAnalyzer^™^ tool. G4 sequences were folded by cooling the samples prepared in KCl (20mM sodium phosphate (referred to as NaPi hereafter), pH7.4 and 100 mM KCl) or LiCl buffers (20 mM Tris-HCl, pH7.4 and 100 mM LiCl) using a thermocycler with 1°C/min. The folded G4 quadruplexes were stored at 4 °C for immediate use or at −20°C for future use. All the chemicals and reagents used in this study were commercially purchased with >98% purity and used without further purification.

### Purification of recombinant proteins

The plasmids, containing a codon-optimized human SERF2 gene, were synthesized commercially by GenScript and subcloned to a pER28a-SUMO vector as reported elsewhere.^42^ The expression and purification of the SERF2 wild-type or different lysine-alanine mutant proteins was like that previously reported for yeast SERF.^42^ Briefly, the plasmids were transformed into competent BL21(DE3) *Escherichia coli* cells, incubated overnight in 10 mL of cell culture medium, and then transferred to freshly prepared 1 L of PMEM medium containing 50 mg/L Kanamycin. The cells were further grown at 37°C, under shaking, until the OD_600_ reached 1.0. Then they were transferred to a 20°C shaker for 1 hour, protein expression was induced by adding 0.1 mM IPTG, and the cells were incubated overnight at 20°C with continued shaking.

Isotope-labeled 15N and 15N-13C SERF2 proteins for NMR studies were produced by growing cells in M-9 minimal media supplemented with 100% 15N NH_4_Cl (1g/L) for the ^15^N labeling or 15N NH_4_Cl and D-Glucose-13C_6_ (4 g/L) for the 15N-13C labeling. Cells were subsequently harvested and lysed by sonication in ice-cold lysis buffer (40 mM Tris-HCl pH 8.0, 10 mM NaPi, 400 mM NaCl, 20 mM imidazole, 10% glycerol, 1 tablet of cOmplete protease inhibitor (Roche), and 1.25 μg/mL DNase I (Roche). The lysate was centrifuged at 36,000 g for 30 minutes and the supernatant was passed through a HisTrap column (Cytiva, 17–5248-02). Lysis buffer containing 0.5 M imidazole was used to elute the His-SUMO tagged SERF2 proteins, the elution was then supplemented with beta-mercaptoethanol to a final concentration of 5mM. The sample was next incubated overnight at 4°C with 10 μL of homemade SUMO protease 6His-ULP1 for His-SUMO cleavage. The digestion mixture was dialyzed in 40 mM Tris-HCl pH8.0 and 300 mM NaCl overnight at 4°C, using a 3.5 kD cutoff dialysis membrane (Repligen, 132724). The dialyzed proteins were run through a 5 mL HisTrap column to remove the cleaved His-ULP1 and the His-SUMO. The flow-through SERF2 protein was further purified by an ion exchange HiTrap SP column (Cytiva, 17–5161-01) using buffer A (50 mM NaPi and 125 mM NaCl pH6.0) and buffer B (50 mM NaPi and 1 M NaCl, pH 6.0). A final purification of the ion-exchange purified SERF2 protein was conducted using a size-exclusion chromatography column Hiload75 (Cytiva, 28989333) in 20 mM NaPi pH7.5 and 150 mM NaCl or 40 mM HEPES pH 7.5 and 100 mM NaCl. The protein samples used in the biophysical and biochemical studies were prepared in indicated buffers as needed via buffer exchange using a 3 kD cutoff filter (EMD Millipore, UFC503024). The SERF2 protein concentration was determined using a Pierce TM BCA assay calibrated with the SERF2 A51W mutant serving as a standard. A similar expression and purification protocol, as described above, was used to produce different SERF2 cysteine and alanine mutants.

For the high throughput screening assay SERF2 protein was prepared containing GST and streptavidin binding peptide (SBP) tags. SERF2 was cloned into a pGEX-6P-1 construct containing GST and SBP tags and transformed into Rosetta-competent cells. Cell cultures were grown in LB at 37 °C until OD_600_ reached ~0.6, thereafter induced with 0.5 mM IPTG overnight at 16 °C. Cells were harvested at 4000×g for 13 minutes and resuspended in lysis buffer (1% triton x-100, 5 mM DTT, 4 mM MgCl_2_, 200 mM NaCl, 20 mM HEPES and 1 tablet/L culture Pierce^™^ Protease Inhibitor Mini Tablets EDTA-free). Lysates were sonicated and incubated with 3 units/L culture RQ1 DNAse (Promega) and 500 units/L culture Benzonase Nuclease (Sigma-Aldrich) for 15 minutes at room temperature. Following incubation, lysates were centrifuged at 17500 rpm for 30 minutes and the supernatant was passed through a 0.45 μm filter. GST-SBP-SERF2 was purified using Pierce^™^ Glutathione Agarose (Thermo Fisher Scientific). Protein was further cleaned up using HiTrap^®^ Heparin High Performance (Sigma Aldrich) and concentration was assessed using Pierce 660 nm assay (Thermo Fisher Scientific). Protein purity was determined by SDS-PAGE and Coomassie blue staining.

### Protein labeling

Cy-5 (Cytiva, PA25031) or Alexa Fluor 488 (AF488) (Invitrogen, A10254) labeling of protein was done by incubating 200 μM of SERF2 (T2C) or G3BP1 with a 10-molar excess of C_5_ maleimide mono-reactive dye in 20 mM Tris-HCl pH7.4 and 100 mM KCl buffer overnight, at 25 °C (SERF2) or at 4 °C (G3BP1), under continuous shaking at 300 rpm. The free excess label was then removed by passing the sample through a PD-10 desalting column in a dark room. The samples were concentrated using a 3 kD Amicon Ultra-15 Centrifugal Filter Unit (EMD Millipore, UFC800324), and any residual free dyes were removed by resuspending the proteins in a working buffer after centrifugation at 8,500 rpm (8 times) for 15 minutes using an Amicon ^®^Ultra 0.5 mL 3 kD cutoff filter.

### High-throughput screening assays

#### RNA bind-n-seq assay and analysis

For the RNA bind-n-seq assay, a single-strand DNA library containing a randomized 40 nucleotide region was obtained from IDT, gel-purified and the RNA library was prepared following a previously described method^55^ using a T7 promoter and in vitro transcription. A second pool of RNA was made by replacing the guanines with 7dG (Trilink) to eliminate RNA G4 quadruplex folding while preserving the sequence. Residual DNA was removed with DNase I (Promega) followed by a phenol-chloroform extraction. The RNA was resolved in a 6% TBE-Urea gel, the expected size band was excised, and gel purification was conducted as previously described.^55^

The RNA bind-n-seq method was modified from a previous study as described below.^55^ Briefly, 60 μL of recombinant GST- SERF2 at different concentrations (250 nM and 50 nM) in binding buffer (25 mM Tris-HCl pH 7.5, 150 mM KCl or LiCl, 3 mM MgCl_2_, 500 μg/mL Ultrapure BSA and SUPERase-In RNase Inhibitor) were equilibrated with 60 μL of pre-washed (binding buffer) magnetic beads (Dynabeads MyOne Streptavidin T1, Invitrogen) for 30 minutes at 4°C. RNA pools were heated in the presence of 150 mM KCl or LiCl for 5 minutes at 100°C and cooled down to room temperature for at least 10 minutes. 60 μL of 3 RNA pools (KCl, LiCl, and 7dG (in LiCl)) were then mixed with GST-SBP-SERF2 and further incubated for 1 hour at 4°C. The final concentrations of protein in the binding reaction were 250 nM and 50 nM and the RNA’s concentration was 1 μM. The protein-RNA complexes were washed with buffer (25 mM Tris-HCl pH 7.5, 150 mM KCl or LiCl, and SUPERase-In RNase Inhibitor (Invitrogen)). The complexes were magnetically isolated, and RNA was eluted with buffer (4 mM biotin and 25 mM tris-HCl pH 7.5) for 30 minutes at 37°C. The elution was performed twice, the eluates were combined, and RNA was purified by a phenol-chloroform method.^55^ Before reverse transcription, RNAs were heated for 5 minutes at 100°C in the presence of 150 mM LiCl to facilitate G-quadruplex unfolding. Following reverse transcription, the samples were prepared for sequencing as described elsewhere.^55^

We performed sequence enrichment analysis as previously described.^55,124^ Briefly, we analyzed k-mer (*k*=6) enrichments to determine ‘R’ values. R was defined as the frequency of a given k-mer in the protein-associated pool divided by the frequency of that *k*mer in the input pool. To specifically determine the enrichment of G quadruplexes, we searched for strong G4 patterns (G_(3–6)_N_(0–7)_)_4_. As a control, we removed all sequences that matched the G4 pattern but still had greater than 8 G’s in the randomized region. The G4 pattern analysis was performed on the randomized region plus the adapters (as described in Reference^55^ for RNA structure analysis), as they are part of the RNAs presented to the protein in the binding reaction. The enrichment of each pattern was the frequency of the pattern in the protein-bound sample divided by its frequency of the input pool.

#### FOREST assay

Library-1 from the previously published paper^56^ which includes 1800 pre-miRNA and 10 RNA G4 quadruplex sequences was used for FOREST screening. A detailed method for oligonucleotide template pool, DNA barcode microarray design, in vitro transcription, RNA fluorophore labeling, hybridization, and microarray scanning has been provided in the supporting information. Briefly, the templates used were synthesized by oligonucleotide library synthesis and the size was limited to 170 nucleotides (OLS, Agilent Technologies). The in vitro transcribed RNA structure library was labeled with Cy5, at the 3’ end, to detect and quantify RNA probes on a microarray. Lastly, the library was prepared in K^+^ folding buffer (10 mM Tris-HCl pH 7.5, 100 mM KCl), heated at 95 °C and cooled to 4 °C at a rate of −6 °C/s on a ProFlex Thermal Cycler (Thermo Fisher Scientific) to allow for G4 folding.

His-tagged SERF2 recombinant protein was used for the FOREST binding assay. For this purpose, the target protein (100 pmol of SERF2), 20 μL of TALON magnetic beads (Clontech), and 1 μg of the refolded RNA structure library were mixed in 1 mL of protein-binding buffer (10 mM Tris-HCl pH 7.5, 100 mM KCl, 10% glycerol, and 0.1 μg/μL BSA). A mixture containing no protein was also prepared as a control. The mixtures were incubated on a rotator at 4 °C for 30 min and washed three times with the protein-binding buffer. Then, 200 μL of elution buffer was added to the magnetic beads, and the mixture was heated at 95 °C for 3-min. The RNA was collected from the supernatant by removing the magnetic beads. The RNA structure library was extracted with phenol and chloroform and an ethanol precipitation was conducted for RNA purification. The enriched RNA sample was hybridized for microarray analysis as detailed in the supporting methods. To determine the protein-binding intensities of each RNA probe, we subtracted the fluorescence intensities of the negative control sample (samples without protein) from those of the enriched protein samples. To account for any undesired interactions with the barcode region, we calculated the average fluorescence intensity of each structure by averaging the intensities of the RNA probes that had the same RNA structure but differing barcodes.

### NMR experiments

NMR samples were prepared in NaPi buffer containing 92%/8% H_2_O/D_2_O. Isotopically unlabeled protein and oligonucleotide samples were used to collect 1D proton NMR spectrum with a recycle delay of 1 second. Multidimensional NMR data were collected using uniformly isotope-labeled 15N or 15N/13C SERF2 samples. A series of 2D and 3D NMR experiments, that included 15N-HSQC,13C-HSQC, HNCO, HNCA, HNCOCA, HNCACO, CBCA(CO)NH, HNCACB, 15N-HSQC-NOESY, and 15NHSQC-TOCSY, were conducted for backbone assignments of 1.0 mM proteins dissolved in 20 mM d_3_-NaAc (sodium acetate) with pH 5.5 and 8% D_2_O at 4 °C. Unless indicated, all NMR measurements were done with protein or protein-RNA mixture samples that had been suspended in 20 mM NaPi and 100 mM KCl (pH 7.4) containing 8% D_2_O at 4 °C. 2D 15N/1H TROSY experiments were recorded for 100 μM 15N labeled SERF2 titrated with an increasing concentration of rG4 for binding site analysis. The effect of pH and temperature on SERF2’s chemical exchange was studied by acquiring the 2D spectrum at 4 and 37 °C, in buffers containing either 20 mM NaPi and 100 mM KCl (pH7.4) or 20 mM d_3_-NaAc (pH 5.5). 15N relaxation NMR measurements, including heteronuclear NOE, T1, and T2, were done using 200 μM 15N SERF2 mixed with and without 100 μM of TERRA rG4. The relaxation delays used for the T1 experiments were 20, 50, 90, 130, 200, 320, 450, 580, 750, 900, 1200, and 2005 ms. T2 relaxation delays used were 16.96, 50.88, 84.80, 135.68, 169.60, 220.48, 305.28, 373.12, 457.92, 542.72, 678.40, and 1356.80 ms. Saturation transfer difference (STD) NMR spectra were recorded with 512 scans, 4 s saturation time, on-resonance excitation at different chemical shift regions, and off-resonance excitation at −40.0 ppm. 2D TROSY, 3D, and STD NMR data were collected on a Bruker 800 MHz spectrometer equipped with a triple resonance cryoprobe. 15N relaxation data were collected on a Bruker 600 MHz equipped with a triple resonance cryoprobe. The NMR data was processed using Bruker’s Topspin 4.1.4 and spectra assignment and analysis was done using an NMRFAM-Sparky 1.47.

### Fluorescence polarization and anisotropy assay

Fluorescently labeled 6-FAM RNA probes were prepared in nuclease-free water containing 20 mM NaPi (pH 7.4) and 100 mM KCl. Fluorescence polarization assays were done using a 20 nM RNA 6-FAM RNA probe mixed with increasing concentrations of SERF2, ranging from 0.009 μM to 20 μM, all dissolved in 20 mM NaPi and 100 mM KCl (pH7.4). The sample mixture was incubated for 30 minutes at room temperature. Fluorescence polarization data were recorded on a TECAN Infinite M1000 microplate reader at 25 °C with the excitation and emission wavelengths set at 470 and 530 nm, respectively. Fluorescence anisotropy measurements were done by titrating SERF2 to 200 nM of Cy3 (excitation/emission, 550/570 nm) or FAM (excitation/emission, 493/517 nm) labeled oligonucleotides, in a 1 mL quartz cuvette (Hellma,101-QS), using a Cary Eclipse spectrofluorometer (Agilent) at 25 °C. Slit bandwidths were set at 5 nm and 10 nm for excitation and emission, respectively. The binding constant (K_D_) was calculated from the change in polarization or anisotropy values, in GraphPad Prism 9.5.1, using non-linear regression for curve fitting with a one-site specific binding model.

### Circular dichroism spectroscopy

The secondary structure of the G4 quadruplexes (15 μM) or SERF2 (50 μM), suspended in 20 mM NaPi and 100 mM KCl (pH7.4), were studied by collecting CD spectra using a JASCO J-1500 spectropolarimeter. For folding analysis, the CD spectra of SERF2 were collected at different temperatures (4, 25, and 37 °C). CD titration was done for 20 μM TERRA rG4 against an increasing SERF2 concentration (10 to 100 μM) to monitor rG4 secondary structure change at 25 °C. The buffered CD spectrum was subtracted from the average CD spectrum obtained from 8 scans.

### Liquid-liquid phase transition assay

16-well Culture-Well chamber slips (Grace Bio-Labs) or 384-well plates (Cellvis, P384–1.5H-N), pre-treated with 5% (w/v) Pluronic^™^ F-127 (Sigma, P2443) overnight, were used to study in vitro phase separation. The well chambers were washed three times with NaPi buffer and air dried. 50 or 20 μl aliquots of reaction sample mixtures were incubated for 30 minutes at room temperature in various conditions (varying protein, total RNA from HeLa cells, poly-ribonucleotides, and rG4 quadruplex concentrations) and a buffer with or without 10% of PEG8000 (Sigma, P5413). Phase transition of G3BP1 was measured in a non-crowding (20 mM NaPi, 100 mM KCl, pH 7.4) or crowding condition (20 mM NaPi, 100 mM KCl, pH 7.4 containing 2.5% or 5% PEG8000) at varying G3BP1, HeLa cells extracted total RNA, SERF2, and rG4 quadruplex concentrations. For fluorescence imaging, 1/200^th^ fluorescence labeled (6-FAM or Cy-5) protein/rG4 quadruplex samples were mixed into unlabeled sample mixtures. Sample imaging and FRAP measurements were done, using a Nikon Ti2-E motorized, inverted microscope. This microscope is controlled by NIS Elements software and contains a SOLA 365 LED light source and a 100X oil immersion objective. Recovery half-life analysis was done using GraphPad Prism and the image processing was done using Fiji ImageJ.

### Mutational analysis

#### Electrophoretic mobility shift assay

5 μM of TERRA quadruplexes, dissolved in NaPi buffer, were mixed with equimolar concentrations of wild-type and different lysine-alanine mutant SERF2 and incubated for 30 minutes at room temperature. Gel shift mobility assays were performed by loading a 5 μM TERRA sample mixture, containing SERF2 and 20% glycerol, to a 4–20% TBE gel (Invitrogen, EC6225).

#### Phase transition assay

50μM of TERRA rG4 quadruplexes was dissolved in 20 mM NaPi, 100 mM KCl, pH 7.4 containing 10% PEG8000 and mixed with different lysine-alanine SERF2 mutants at equimolar concentration. The samples were incubated for 30 minutes at room temperature and DIC images were taken on a Nikon Ti2-E motorized, inverted microscope.

### Size-distribution analysis

#### Size-exclusion chromatography

20 μM of TERRA quadruplexes, dissolved in NaPi buffer, were mixed with 40 μM SERF2 and incubated for 30 minutes at room temperature. The mixture was then injected into a Superdex 200 Increase 10/300 GL size-exclusion chromatography column (Cytiva, 28–9909-44).

#### Analytical Ultra Centrifugation (AUC)

The AUC measurements were done for a SERF2 (9.4 μM) and TERRA rG4 quadruplex (4.7 μM) sample mixture that was dissolved in 20 mM NaPi, 100 mM KCl (pH 7.4) at 22 °C. AUC measurements were done at 260 nm, where SERF2 has no absorption, and with an intensity mode of 42,000 rpm. 420 μL samples were loaded into a two-channel epon-charcoal centerpiece, with a 1.2 cm path length, in an An60Ti rotor of a Beckman Optima Xl-I AUC. The data were analyzed with Ultrascan III software (version 4) and the LIMS server using the computing clusters available at the University of Texas Health Science Center and XSEDE sites at the Texas Advanced Computing Center.

### Structure calculation and all-atom MD simulations

SERF2 backbone and NOE assignments were done using NMRFAM-Sparky and the dihedral angles were predicted utilizing the backbone chemical shifts by the TALOS-N program.^80^ A total of 494 NOE distance constraints were used for the multiple-state ensemble calculation in CYANA 3.98.15. One hundred conformers were calculated using 10,000 torsion-angle dynamic steps. The 20 conformers, with the lowest target function values, were used to represent the calculated SERF2 structure and subjected to refinement using Crystallography & NMR System (CNS) energy minimization in an explicit water model. The CYANA-generated SERF2 PDB coordinates, NOE constraints, dihedral angles, and hydrogen bond constraints were input to CNS for structure refinement.

The MD simulations were performed using GPU nodes running in parallel. To gauge the intrinsic structural dynamics of these SERF2-TERRA systems, we performed all-atom MD simulations, that were defined in a structure-based balanced forcefield, i.e., CHARMM36m using GROMACS 2022.4.^125^ The SERF2 structure was initially subjected to a 100 ns all-atom MD simulation in CHARMM36m force-field at pH 7.4 and 0.1M KCl for structural refinement. The SERF2-TERRA complex structure was next built, using the HADDOCK program,^83^ by parsing the NMR distance restraints obtained from chemical shift perturbations and saturation transfer NMR data analysis. A set of ambiguous active site SERF2 residues (T3, N5, R7, R11, Q12, K16, S19, S21, K23, A33, Q46, K47, A51, N52, K55 and E56) and TERRA guanines (G5, G9, and G10) were provided to the HADDOCK program to allow it to build the structure of the complex. The energetically best cluster complex structure was used for MD simulation analysis by solvating in the TIP3P water model in an orthorhombic water box. These model systems were electro-neutralized using 100 mM KCl followed by energy minimization, using the steepest descent algorithms in less than 5000 steps, to remove steric clashes. The energy-minimized systems were then subjected to two-step equilibrations using NVT and NPT ensembles for 50 ns and keeping the temperature to 37 °C and pressure to 1 bar using a V-rescale thermostat and Berendsen barostat. These equilibrated systems were subjected to a final production MD of 0.5 μs. All the bonds involving hydrogen atoms were restrained using the SHAKE algorithm. The atomic coordinates of each system were saved every 100 ps resulting in 5000 snapshots for post-dynamics analysis.

### MD simulation of SERF2-TERRA liquid-liquid phase separation

We designed two densely packed, multi-component systems, named small and large, to study SERF2 and TERRA rG4 condensates using atomistic MD simulation. The MD system comprised randomly distributed multiple copies of SERF2, TERRA, and 10% PEG8000 molecules solvated in a TIP3P water model in an orthorhombic water box with the dimensions of 20nm×20nm×20nm. To mechanize the complex molecular assembly and preparation of the input files for the MD simulation under periodic boundary conditions, we used a Multicomponent Assembler in CHARMM-GUI.^126^ The small system consisted of 6 SERF2, 6 TERRA, 60 units of PEG8000, 0.1 M KCl (234 K^+^ and 60 Cl^−^), and 23185 water molecules. The large system contained 30 units of SERF2, 30 TERRA rG4, 150 PEG8000, 0.1 M KCl (1266 K^+^ and 396 Cl^−^), and 216499 water molecules. The input files for the MD simulations were generated using a CHARMM36m force field compatible with GROAMCS. The same MD simulation procedure involving energy minimization, two-step equilibration, and production was adopted, as described above, for both densely packed systems. The small system was subjected to 1 μs production MD, while the large systems were set to 0.5 μs. The trajectories of both systems were harvested at 100 ps intervals for post-dynamics analysis.

### Post-dynamic analysis of MD trajectories

We employed the Gromos Clustering Algorithm method, with a cut-off of 0.2 nm, to obtain a representative conformation of the entire trajectory with maximum population. The hydrogen bond occupancy of each residue was computed using the hydrogen bonding analysis toolkit of Visual Molecular Dynamics (VMD 1.9.4a53). An angle cutoff of 30° and donor-acceptor distance of 3.5 Å was set to compute hydrogen bonds between protein and nucleic acids. The residue pairs (amino-acid and nucleic acid) having higher percentages of hydrogen-bond occupancy were used for the generation of interaction plots. Structural representations were created using PyMOL and the BIOVIA Discovery Studio Visualizer. To probe the diffusion of SERF2 molecules, in the small and large MD systems, the mean square displacement of individual SERF2 molecules was calculated. The average end-to-end distance between the nitrogen atoms of T2 and A51 in SERF2 was computed using the 0.5 μs MD trajectory.

### Single-molecule fluorescence microscopy and droplet fusion by optical tweezers

Single particle tracking (SPT) and fluorescence resonance energy transfer (FRET) experiments were conducted using a double cysteine SERF2 mutant (A2C and A51C) labeled with Cy3 and Cy5 fluorophores. 200 μM of double-mutant SERF2 (A2C, A51C) was fluorescently labeled by incubating it with 5x molar excess of Cy3 and Cy5, applying the same protocol described above in the section ‘Protein labeling’. The occupancy of the Cy3 and Cy5 in each SERF2 molecule was not determined because the SPT and FRET measurements do not rely on a uniform 1:1 Cy3:Cy5 labeling for studying SERF2-rG4 condensates. For single-molecule experiments, droplets were prepared by mixing 100 μM unlabeled SERF2 and TERRA containing a picomolar concentration of Cy3-Cy5 SERF2 or diluted as needed to obtain a sparse concentration where single molecules are distinguishable for SPT. The sample chamber was prepared by mounting #1.5 coverslips on a standard glass microscope, using double-sided tape placed about 5mm apart as spacers. The chamber was coated with ~15 μL of 0.1 mg BSA (5 minutes) followed by rinsing thrice with NaPi buffer containing 10% PEG8000. SERF2-TERRA droplets were then injected into the sample chamber and droplets were allowed to settle for ~20 minutes. An O_2_-scavenging system (1 mM trolox, 2.5 mM protocatechuic acid (PCA), and 7 μg/mL protocatechuate-3,4-dioxygenase (PCD)) prepared in NaPi, PEG8000 buffer was then applied to the sample chamber immediately before performing the SPT and FRET measurements at room temperature. Fluorescent images were acquired with total internal reflectance fluorescence (TIRF) illumination (using an Olympus CellTIRF module). Acceptor and donor fluorescent images were collected simultaneously on dual Andor Ultra EMCCD cameras at frame rates between 10Hz and 100Hz. The data were analyzed using an in-house MATLAB script to obtain the diffusion constants via a linear fit of the mean squared displacement for each particle and FRET efficiency. The fusion of SERF2 and rG4 droplets was studied using a bespoke dual-trap optical tweezers instrument equipped with a bright-field camera and a 60X oil immersion objective (Nikon APO TIRF 60X). Two optical traps are generated by splitting a 10W 1064nm laser by polarity, with each polarized beam steered by an acousto-optic deflector (IntraAction DTD-274HD6). Each particle was trapped using approximately 250 mW laser power. 100 μM of SERF2 was mixed with equimolar 23- or 12-nucleotide long TERRA rG4s in NaPi buffer containing 10% PEG8000 (v/v). The glass slide chamber was prepared as described above and coated with 0.1 mg BSA (5 minutes) followed by rinsing. 15 μL of SERF2-TERRA droplet samples were passed into a glass slide chamber. Particle fusion was observed by trapping two condensate droplets in separate optical traps and steering the laser foci together until particle fusion was observed. Movies of fusion events were recorded within ~5 minutes at room temperature to avoid droplet settlement on the glass slide and droplet aging. The droplet fusion time was extracted using the ImageJ program.

## Supplementary Material

1

## Data Availability

Atomic coordinates for human SERF2 have been deposited in the RCSB Protein Data Bank (PDB) with accession numbers 9DT0.
