# Supplementary material for: Visualizing liquid-liquid phase transitions
Source: bioRxiv. 2024 Oct 28:2023.10.09.561572. Preprint. [Version 2] doi: 10.1101/2023.10.09.561572 (PMC11565804; doi:10.1101/2023.10.09.561572)
Supplement: Supplementary file 1 [file NIHPP2023.10.09.561572V2-supplement-1.pdf]

bioRxiv preprint doi: https://doi.org/10.1101/2023.10.09.561572; this version posted October 28, 2024. The copyright holder for this preprint (which was not certified by peer review) is the author/funder, who has granted bioRxiv a license to display the preprint in perpetuity. It is made available under aCC-BY 4.0 International license.

Supplementary Data Figures

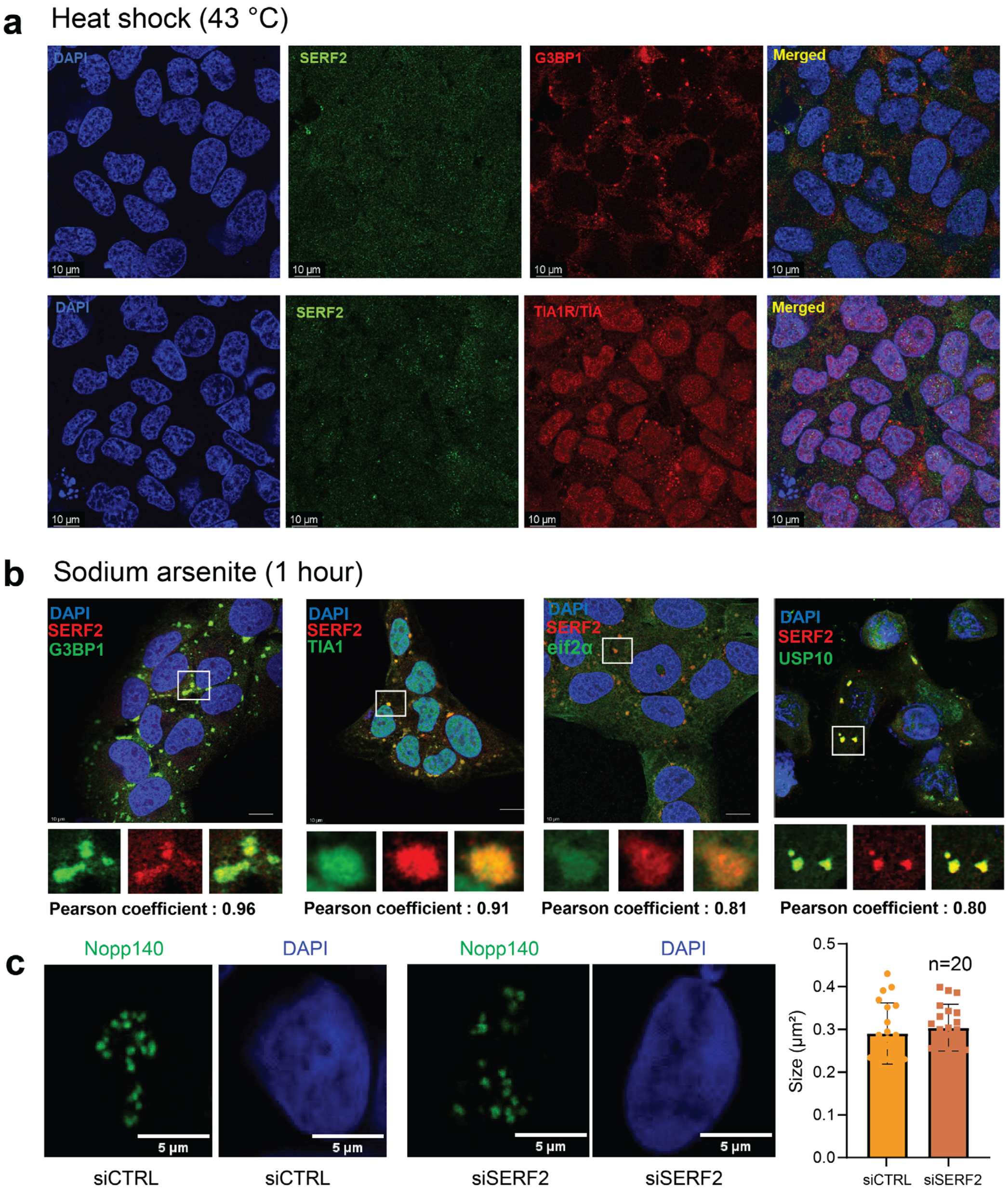

Supplementary Data Fig. 1 | SERF2 colocalized with different stress granule proteins. **a,** Immunofluorescence of SERF2, G3BP1 and TIA1 in fixed U2OS cells treated with heat shock. **b,** Immunofluorescence of SERF2, G3BP1, TIA1, eIF2α and USP10 in fixed U2OS cells treated with sodium arsenite. Comparing the size and shape distribution of nucleolus in cells treated with 0.4M sorbitol in siControl and siSERF2 conditions. Fixed cells were treated respectively with Nopp140 and DAPI to mark the nucleolus and nucleus.

bioRxiv preprint doi: https://doi.org/10.1101/2023.10.09.561572; this version posted October 28, 2024. The copyright holder for this preprint (which was not certified by peer review) is the author/funder, who has granted bioRxiv a license to display the preprint in perpetuity. It is made available under aCC-BY 4.0 International license.

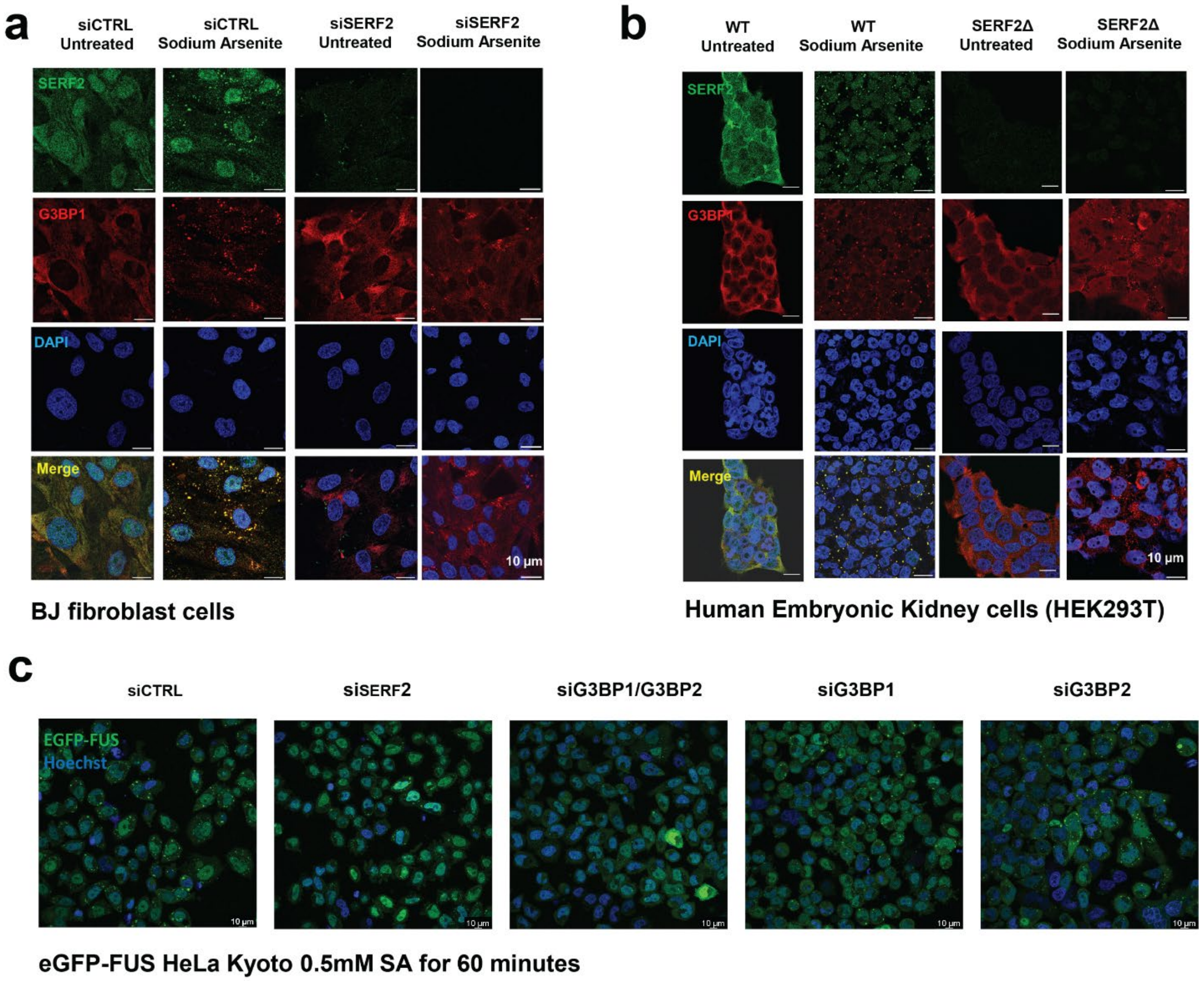

Supplementary Data Fig. 2 | SERF deletion or knockout affects stress granule assembly. a, Immunofluorescence of SERF2 and G3BP1 in fixed BJ fibroblast cells with siCTRL or siSERF2 knockdown. **b,** Immunofluorescence of SERF2 and G3BP1 in fixed HEK293T WT and SERF2 deletion cells. **c,** Live cell imaging of EGFP-FUS HeLa Kyoto cells with siCTRL, siSERF2, siG3BP1/G3BP2, si G3BP1 or si G3BP2 knockdown. Cells were treated with 0.5mM sodium arsenate for 30 minutes before imaging.

bioRxiv preprint doi: https://doi.org/10.1101/2023.10.09.561572; this version posted October 28, 2024. The copyright holder for this preprint (which was not certified by peer review) is the author/funder, who has granted bioRxiv a license to display the preprint in perpetuity. It is made available under aCC-BY 4.0 International license.

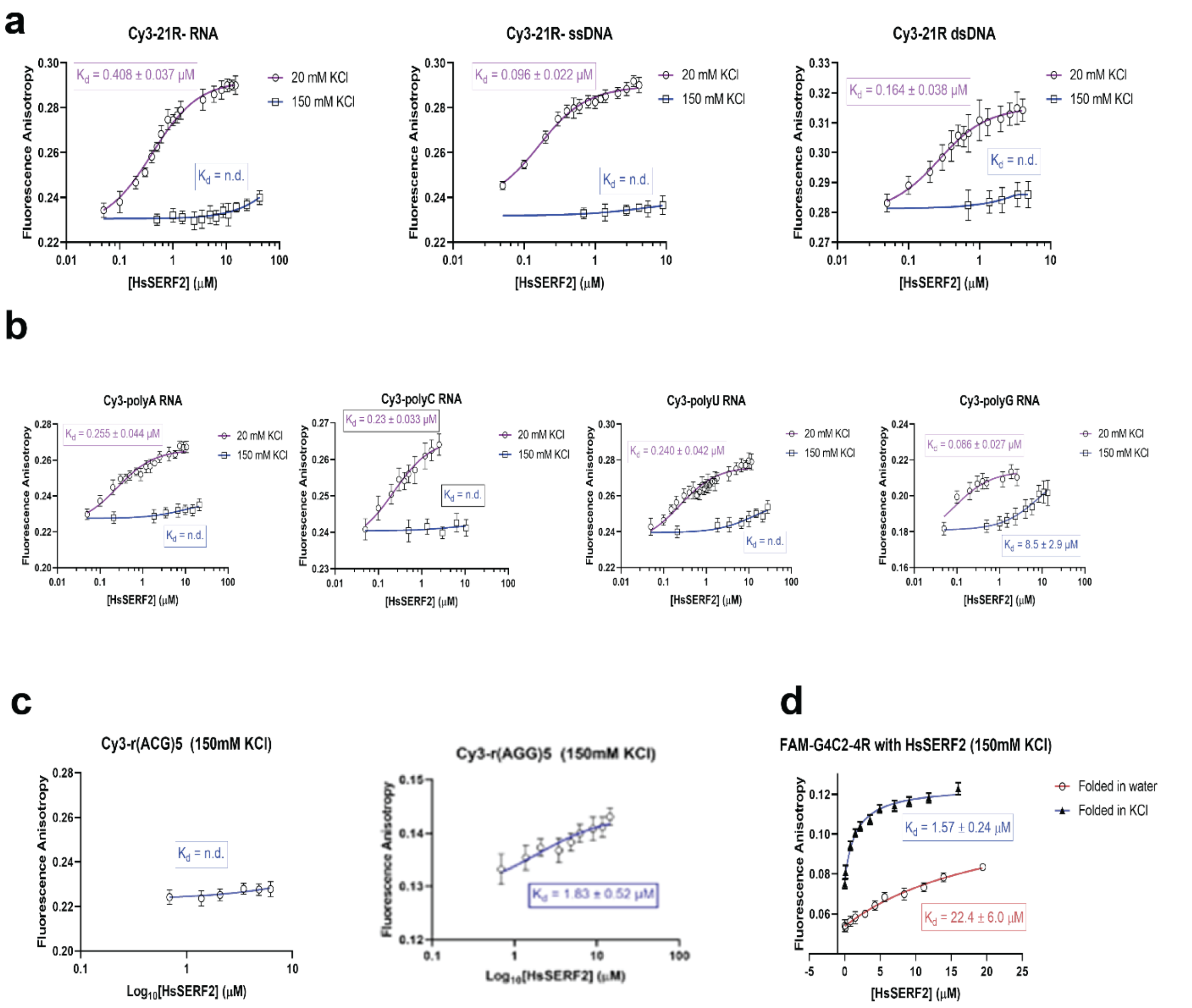

Supplementary Data Fig. 3 | SERF2 binds specifically to folded RNA G-quadruplexes. Fluorescence anisotropy titration of human SERF2 protein into Cy3 labeled 21R- single-stranded RNA, single-stranded DNA and double-stranded DNA (**a**); Cy3 labeled poly-tract RNAs with 21 nucleotides in length (**b**); Cy3 labeled (ACG)5 and (AGG)5 RNA (**c**), and 6FAM labeled 4-repeat G4C2 (**d**).

bioRxiv preprint doi: https://doi.org/10.1101/2023.10.09.561572; this version posted October 28, 2024. The copyright holder for this preprint (which was not certified by peer review) is the author/funder, who has granted bioRxiv a license to display the preprint in perpetuity. It is made available under aCC-BY 4.0 International license.

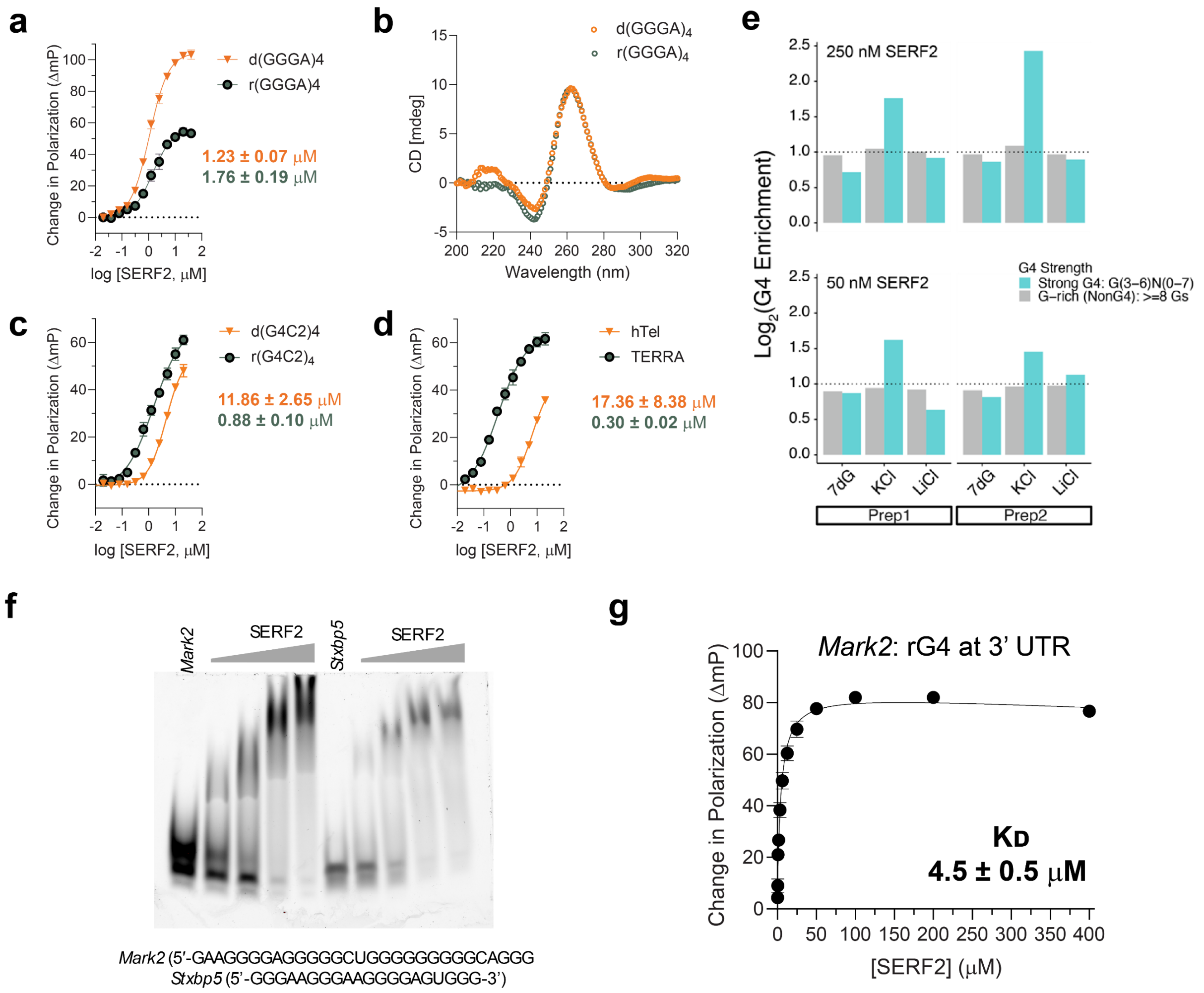

Supplementary Data Fig. 4 | Fluorescence polarization assay comparing the binding affinity of SERF2 between DNA and RNA G-quadruplexes. **a,** FP binding curves of SERF2 with a 4-repeat GGGA DNA or RNA sequence**. b,** Secondary structure of 20 μM DNA or RNA 4-repeat GGGA rG4s dissolved in 20 mM NaPi, 100 mM KCl at 25°C. **c,d,** FP binding curves of SERF2 with 4-repeat DNA versus RNA G4C2 **(c),** and 4-repeat human telomeric repeat sequence TTAGGG versus a 23-nucleotide TERRA RNA. All FP assays were done with an increasing concentration of SERF2 against a fixed 100 nM concentration of DNA or RNA G4 dissolved in 20 mM NaPi, 100 mM KCl at room temperature. The binding constants were calculated by fitting the FP curves using a binding saturation one-site total model in GraphPad Prism. **e**, Enrichment of rG4 quadruplexes in RNA bind-n-seq analysis in two different preparations in RNA pool prepared in KCl, 7-deaza, and LiCl solution. RNA sequences containing 3 or more guanines in the G-tetrad are referred to as strong G4 quadruplexes and sequences with >=8 guanines but lacking a defined G4 quadruplex forming motif are referred to as non-G4s. The enrichment analysis done for two different SERF2 concentrations (250 nM, top; 50 nM, bottom) shows a higher rG4 quadruplex enrichment for the highest (250 nM) SERF2 concentration in the KCl solution. **f**, EMSA gel-shift assay shows binding of SERF2 (5, 10, 20 and 40 μM) to rG4 quadruplexes (10 μM) derived from the 3' UTR regions of *Mark2* and *Stxbp5* genes. **g**, Binding affinity measurement between SERF2 and 100 nM 6-FAM labeled rG4 quadruplex present at 3' UTR in *Mark2* gene.

bioRxiv preprint doi: https://doi.org/10.1101/2023.10.09.561572; this version posted October 28, 2024. The copyright holder for this preprint (which was not certified by peer review) is the author/funder, who has granted bioRxiv a license to display the preprint in perpetuity. It is made available under aCC-BY 4.0 International license.

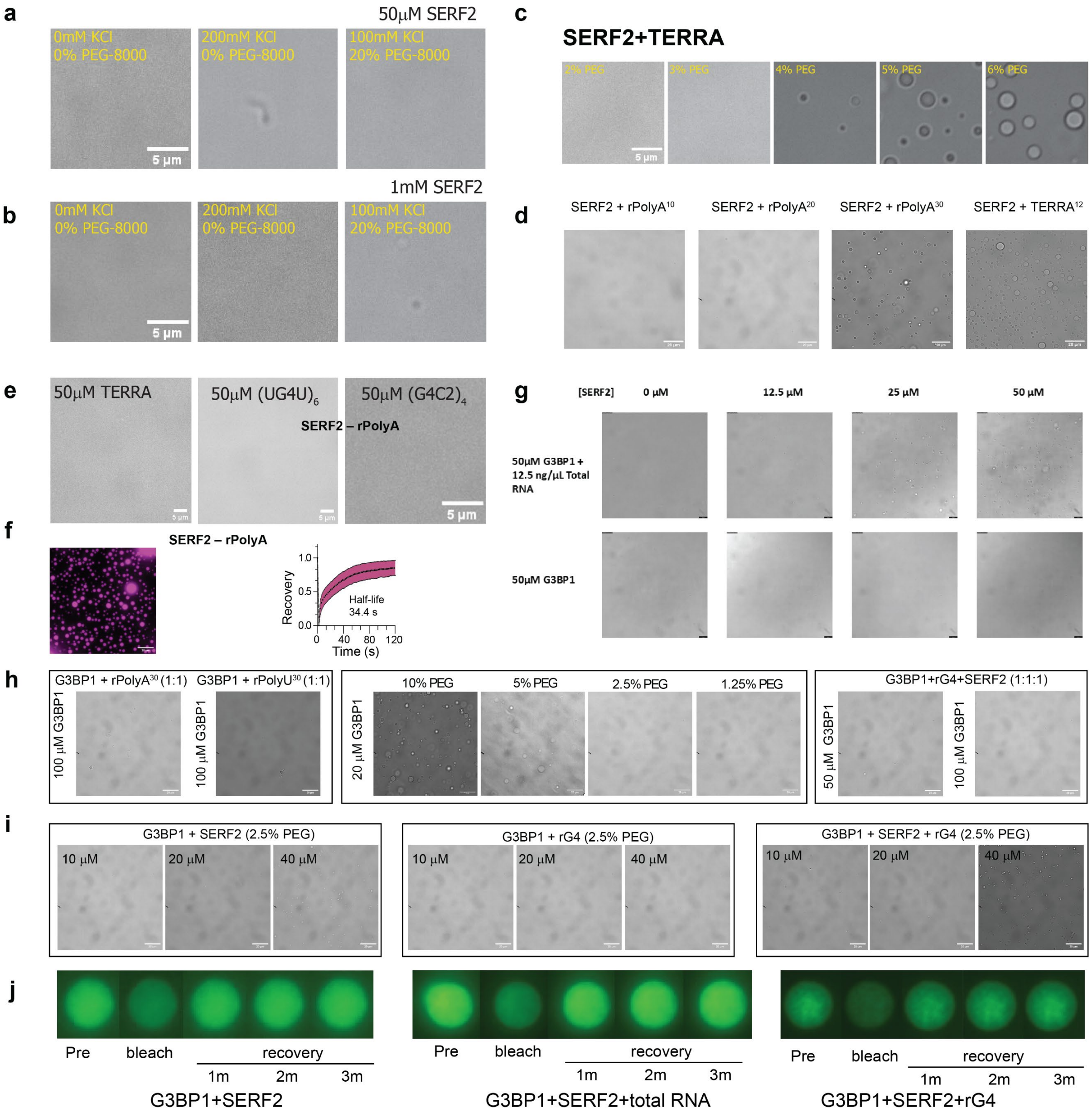

Supplementary Data Fig. 5 |Phase transition property of SERF2. **a,b,** DIC images show no droplet formation for 50 µM **(a)** and 1 mM **(b)** SERF2 dissolved in 20 mM NaPi, pH 7.4 containing different salts and PEG8000 as indicated. **c,** DIC images of 50 µM TERRA mixed with equimolar SERF2 dissolved in 20 mM NaPi, 100 mM KCl, pH 7.4 containing variable PEG8000 as indicated. **d,** Liquid-liquid phase separation in 50 µM SERF2 mixed with 50 µM of 10-nucleotide polyA (left) or 12-nucleotide TERRA (right) in NaPi buffer containing 10% PEG8000 and 100 mM KCl. **e,** Images show all three RNA G4 quadruplexes do not form droplets on their own in 20 mM NaPi, 100 mM KCl, pH 7.4 buffer containing 10% PEG8000. **f,** Phase transition of 50 µM SERF2 mixed with long random-length polyA RNA dissolved in 20 mM NaPi, 100 mM KCl, pH 7.4 buffer containing 10% PEG8000 and 1/200th Cy5-SERF2 (purple signals). The plot on the right shows the average FRAP recovery of SERF2. **g**, DIC images showing

bioRxiv preprint doi: https://doi.org/10.1101/2023.10.09.561572; this version posted October 28, 2024. The copyright holder for this preprint (which was not certified by peer review) is the author/funder, who has granted bioRxiv a license to display the preprint in perpetuity. It is made available under aCC-BY 4.0 International license.

droplet formation in SERF2 mixed with 50 μM G3BP1 and 12.5 ng/μL of total RNA isolated from HeLa cells (top) at the indicated concentration. 50 μM G3BP1 (bottom) shows no droplets under the same conditions in the absence of SERF2 and total RNA. **h,** DIC images showing the phase transition of G3BP1 at the indicated concentration in crowding or non-crowding environments mixed without or with RNA and SERF2. **i,** DIC images show phase transitions of 20 μM G3BP1 suspended in 2.5% w/v PEG8000 mixed with an increasing concentration (10, 20, and 40 μM) of SERF2, 6-repeat UG4U rG4 and the mixture of SERF2 and rG4 quadruplexes. **j,** Time-series FRAP recovery images of SERF2-AF488 in different condensates containing G3BP1, total RNA, or rG4 quadruplexes as indicated. The FRAP recovery plots are shown in Fig. 4m (right panel).

bioRxiv preprint doi: https://doi.org/10.1101/2023.10.09.561572; this version posted October 28, 2024. The copyright holder for this preprint (which was not certified by peer review) is the author/funder, who has granted bioRxiv a license to display the preprint in perpetuity. It is made available under aCC-BY 4.0 International license.

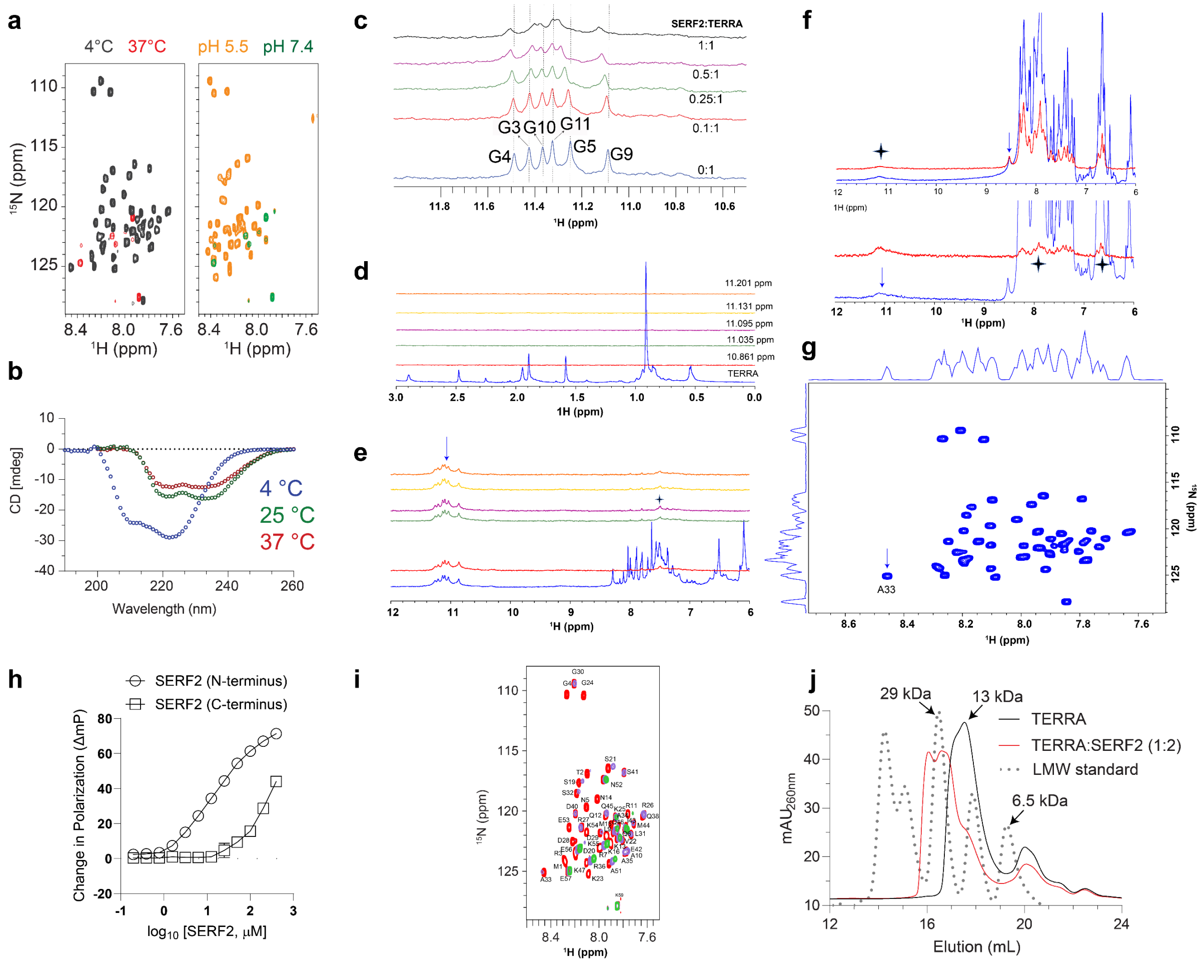

Supplementary Data Fig. 6 | The N-terminal domain in SERF2 is disordered and binds rG4. a, Overlaid 15N/1H TROSY NMR spectra of 100 μM SERF2 recorded at 4 °C (black) or 37 °C (red and green) dissolved in 20 mM NaPi (pH7.4), 100 mM KCl. The missing SERF2 peaks are detectable at 37 °C when dissolved in 20 mM d3-NaAc (pH 5.5),100 mM KCl (orange). The NMR data were collected on a Bruker 800 MHz in buffer containing 8% D_2O. **b,** Secondary structure analysis of human SERF2 by CD spectroscopy at the indicated temperature in 20 mM NaPi (pH 7.4), 100 mM KCl. **c,** Monitoring the stability of imino protons in 200 μM TERRA rG4 quadruplex dissolved in 20 mM NaPi, 100 mM KCl, 8% D2O, pH 7.4 titrated with an increasing concentration of SERF2 by NMR. **d-f,** Saturation transfer difference NMR spectra of 100 μM TERRA without **(d)** and mixed with 100 μM SERF2 **(e,f)**. The saturated TERRA imino proton chemical shift values are labelled in the absence of SERF2 **(d)**. Reference spectrum is shown in blue, and saturated protons/regions are indicated by an arrow and regions showing magnetization transfer are indicated with star-shape. **g,** $^{15}N/^{1}H$ 2D spectrum of SERF2 showing the amide proton of residue A33 do not overlap with TERRA amino and imino signals. The NMR data were collected at 4°C on a Bruker 800 MHz. **h,** FP assay measuring the binding affinity of N-terminal (1-32) and C-terminal (31-59) SERF2 to 100 nM 6-FAM TERRA G-quadruplex. The FP binding data were generated from three independent replicates and curves were fitted by GraphPad Prism non-linear regression curve fit and one-site fitting model analysis. **i,** Overlaid 15N/1H TROSY NMR spectra of 100 μM SERF2 (red) mixed with 50 μM TERRA (purple) and 100 μM TERRA (green). The NMR data were collected at 4 °C on a Bruker 800 MHz. **j,** Size-exclusion chromatography profiles of 20 μM TERRA mixed with 40 μM of SERF2 dissolved in 20 mM NaPi, 100 mM KCl, pH 7.4 buffer. The SEC profile shown in the dashed line corresponds to a low-molecular-weight (LMW) standard.

bioRxiv preprint doi: https://doi.org/10.1101/2023.10.09.561572; this version posted October 28, 2024. The copyright holder for this preprint (which was not certified by peer review) is the author/funder, who has granted bioRxiv a license to display the preprint in perpetuity. It is made available under aCC-BY 4.0 International license.

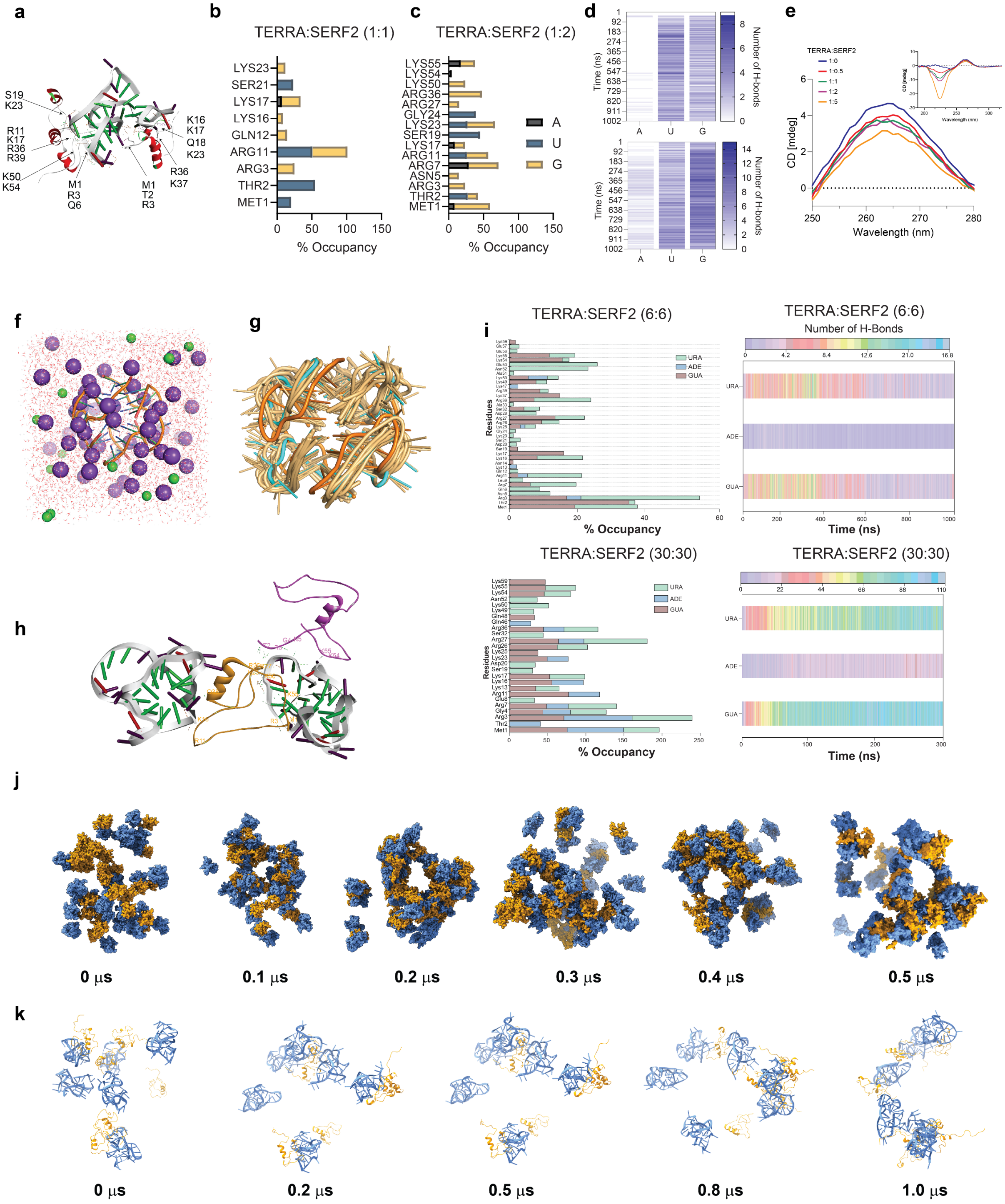

bioRxiv preprint doi: https://doi.org/10.1101/2023.10.09.561572; this version posted October 28, 2024. The copyright holder for this preprint (which was not certified by peer review) is the author/funder, who has granted bioRxiv a license to display the preprint in perpetuity. It is made available under aCC-BY 4.0 International license.

Supplementary Data Fig. 7 | Large-scale all-atom MD simulation of SERF2 and RG4 quadruplex complex. a, Cartoon structure of SERF2-TERRA 2:1 complex shows N- and C-terminal charge residues in SERF2 interact with TERRA rG4 quadruplex. **b,c,** Hydrogen bond occupancy (%) of SERF2 residues in 1:1 (**b**) and 2:1 © SERF2-TERRA complex with individual TERRA nucleotides A/U/G estimated from 0.5 µs MD trajectory using VMD program. **d,** Existence, and disappearance of hydrogen bonds in SERF2-TERRA 1:1 (top) and 2:1 (bottom) complex as a function of nucleotides A/U/G. The scale represents the total number of hydrogen bonds between SERF2 and TERRA in a given time frame. **e,** Monitoring the change in TERRA (20 µM) parallel secondary structure (250-280 nm) by CD spectroscopy in the presence of an increasing amount of SERF2 as indicated. The full CD spectra are shown in the inset. CD spectra were collected at 25 °C for samples dissolved in 20 mM NaPi, 100 mM KCl, pH 7.4. **f,** MD simulation of tetrameric TERRA rG4 (PDB: 2M18) in 150 mM KCl, pH 7.4. The RNA, water molecules, K^{+}, and Cl^{-} atoms are shown in cartoon, red lines, purple and green spheres, respectively. **g,** The cartoon structure shows 10 superimposed structures of TERRA rG4 quadruplex retrieved at 10 ns intervals. The TERRA rG4 structure at 0 ns and 100 ns are shown in dark orange and cyan, respectively. **h,** Interaction network between SERF2 and TERRA 2:2 complex in the dilute phase obtained from 0.5 µs all-atom MD simulations. The SERF2 molecules are shown in orange and pink, and their interacting residues with TERRA molecules are labeled. **i,** Interaction network between SERF2 and TERRA in large-scale all-atom MD simulations. SERF2 residue-specific interaction with TERRA rG4s presented as % occupancy referring to the hydrogen bonds occupancy as a function of simulation time in a small MD system containing 6 SERF2 and TERRA molecules**,** and in a large MD system consisting of 30 SERF2 and TERRA molecules. The number of hydrogen bonds in the corresponding small and large MD systems is shown on the right. The % occupancy over 100% means a single residue forming multiple bonds with the TERRA molecules. **j,** MD snapshots showing the formation of a ring-like structure retrieved at every 100 ns in the large-scale MD simulation system comprising 30 molecules of SERF2 and TERRA rG4 quadruplexes. **k,** Structure of a SERF2 and TERRA complex retrieved at different time points from the 1 µs MD simulation of a small system consisting of 6 molecules of SERF2 (orange) and TERRA (blue) showing oligomerization and early phase of ring-structure formation.

bioRxiv preprint doi: https://doi.org/10.1101/2023.10.09.561572; this version posted October 28, 2024. The copyright holder for this preprint (which was not certified by peer review) is the author/funder, who has granted bioRxiv a license to display the preprint in perpetuity. It is made available under aCC-BY 4.0 International license.

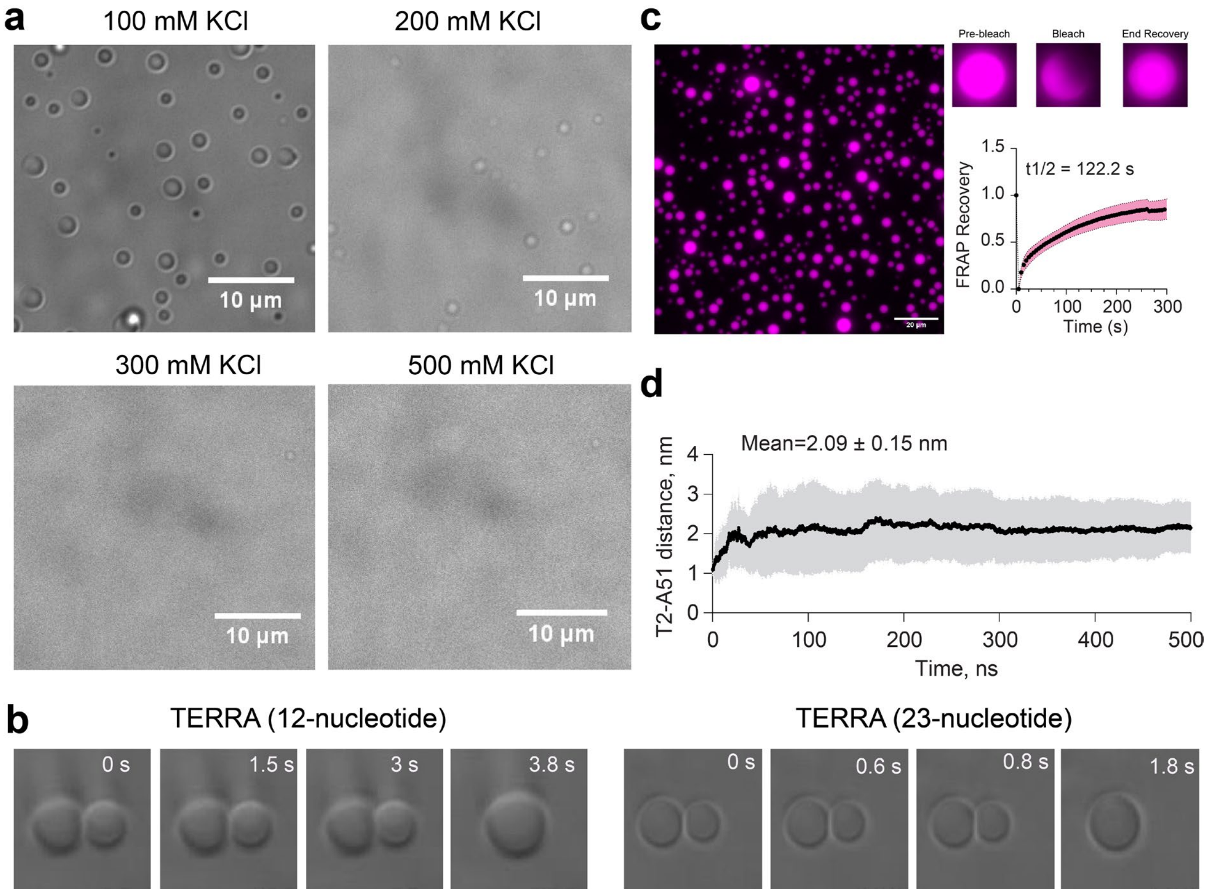

Supplementary Data Fig. 8 |Phase transition of SERF2 and TERRA rG4 quadruplexes. **a,** Effect of salt on SERF2 and 12-nucleotide TERRA phase transition. 50 μM of SERF2 was mixed with equimolar TERRA rG4 in 20 mM NaPi, pH 7.4 containing variable salt as indicated and 10% w/v PEG8000. **b,** Measuring the SERF2 and TERRA rG4 quadruplex phase separated droplet fusion time by a dual-trap optical tweezer. **c,** Phase transition of 100 μM SERF2 containing 1/200th Cy5-labelled SERF2 (purple) mixed with equimolar 12-nucleotide TERRA dissolved in 20 mM NaPi, 100 mM KCl, pH 7.4. The droplet end recovery before and after FRAP is shown on the right. FRAP recovery plot of SERF2 (100 μM) in droplets containing equimolar 12-nucleotide TERRA rG4s (bottom right). The half-time was calculated by fitting the curve using a one-phase association model in GraphPad Prism. **d,** The plot shows the average end-to-end distance calculated from nitrogen atoms of T2 and A51 in each SERF2 molecule in the SERF2-TERRA all-atom MD system comprising 30 molecules of protein and rG4.

Supporting video S1. The video shows the formation of a large condensed-like SERF2-TERRA rG4 quadruplex complex structure and a few small oligomeric structures during 0.5 microseconds all-atom MD simulation. The cartoon representation of SERF2 and RNA molecules is shown in orange and blue, respectively in the VMD program. The black spheres represent the center of mass of each SERF2 molecule in the system. Water, ions, and crowding molecules are removed from the movie generation in VMD.
